# *Lactobacillus* derived small RNAs protect against *Klebsiella pneumoniae* infection

**DOI:** 10.1002/imt2.70184

**Published:** 2026-10-16

**Authors:** Tingting Xu, Xiuying Tao, Dan Fang, Haijie Zhang, Zhiqiang Wang, Yuan Liu

**Affiliations:** ^1^ Jiangsu Co‐innovation Center for Prevention and Control of Important Animal Infectious Diseases and Zoonoses College of Veterinary Medicine, Yangzhou University Yangzhou China; ^2^ Jiangsu Key Laboratory of Zoonosis Yangzhou University Yangzhou China; ^3^ Jiangsu Interdisciplinary Center for Zoonoses and Biosafety Yangzhou University Yangzhou China

**Keywords:** CRKP, gut microbiota, membrane vesicles, natural products, sRNAs

## Abstract

Carbapenem‐resistant *Klebsiella pneumoniae* (CRKP) is a major multidrug‐resistant pathogen with limited treatment options. Plant‐derived natural products have broad biological activities, but their protective effects against extraintestinal multidrug‐resistant bacterial infections remain poorly understood. Herein, we show that selected plant‐derived natural products protect mice against CRKP pulmonary infection by remodeling the gut microbiota. Fecal microbiota transplantation and microbiota‐depletion experiments demonstrated that this protection was microbiota dependent, with *Lactobacillus* emerging as a key genus associated with reduced pulmonary bacterial burden. Among four isolated *Lactobacillus* species, *Lactobacillus murinus* (*L. murinus*) conferred the strongest protection against CRKP infection. Mechanistically, *L. murinus*‐derived membrane vesicles (MVs) delivered the small RNA mim‐miR‐21a‐5p to pulmonary macrophages, where it targeted *PDCD4* and activated the Nrf2/SOD2 antioxidant pathway. This signaling cascade preserved mitochondrial oxidative phosphorylation, promoted an M2‐like macrophage phenotype, and ultimately restricted CRKP infection. Consistent with these findings, intestinal *Lactobacillus*, particularly *L. murinus*, was reduced in patients with CRKP infection compared with healthy controls. Collectively, our findings reveal a mechanism by which plant‐derived natural products enhance host resistance to CRKP through microbiota remodeling and identify *L. murinus*‐derived small RNAs and membrane vesicles as key mediator of this gut‐lung protective axis.

## Highlights

- Plant‐derived natural products protect against CRKP by remodeling gut microbiota, with *Lactobacillus* as a core genus.
- mim‐miR‐21a‐5p in *Lactobacillus*‐derived MVs is a key regulator of macrophage metabolism and anti‐infective immunity.
- *Lactobacillus murinus* (*L. murinus*)‐derived mim‐miR‐21a‐5p specifically targets *PDCD4* in pulmonary macrophages, stabilizes mitochondrial oxidative phosphorylation, and promotes macrophage M2 polarization.

## Graphical abstract

This study reveals that plant‐derived natural products confer protection against carbapenem‐resistant *Klebsiella pneumoniae* (CRKP) infection via remodeling the gut microbiota, with *Lactobacillus* identified as the core beneficial genus. Mechanistically, *Lactobacillus murinus* (*L. murinus*)‐derived mim‐miR‐21a‐5p carried by membrane vesicles (MVs) targets *PDCD4*. This interaction further activates the Nrf2/SOD2 signaling axis, promotes M2 macrophage polarization, and ultimately suppresses CRKP infection.

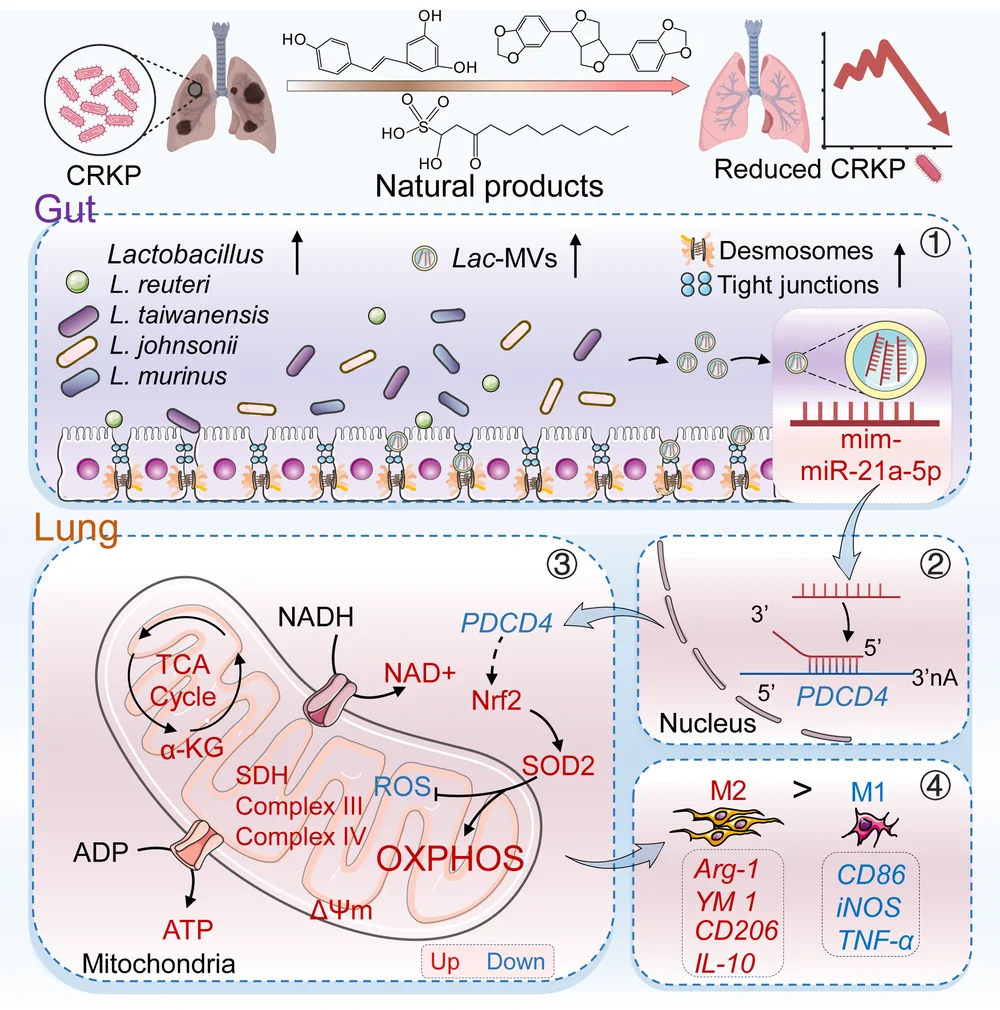

## INTRODUCTION

Carbapenem‐resistant *Klebsiella pneumoniae* (CRKP) is one of the most formidable antimicrobial resistance threats to global public health. CRKP causes diverse multisystem infections, including pneumonia, bloodstream infections, intra‐abdominal infections, and urinary tract infections [1, 2, 3]. Among these, respiratory tract infections constitute the most prevalent clinical manifestation of CRKP infections in China [4]. The detection rate of CRKP in respiratory specimens has surpassed that of other resistant bacteria, positioning it as a critical priority for the prevention and control of clinical respiratory infections [5, 6]. Carbapenem antibiotics serve as the last line of defense against multidrug‐resistant (MDR) Gram‐negative bacterial infections. However, the emergence of carbapenem‐resistant pathogens has precipitated a dire clinical scenario with limited effective therapeutic options. This situation is particularly concerning for CRKP pneumonia, in which treatment failure rates rise markedly owing to concurrent immune dysfunction, rapid bacterial proliferation, and broad antimicrobial resistance [4, 7, 8, 9, 10, 11]. World Health Organization (WHO) has placed CRKP at the top of its 2024 list of priority bacterial pathogens, advocating for enhanced global control measures and underscoring the urgent need to develop distinct treatments for CRKP‐associated infections [12].

Plant‐derived natural products hold considerable promise for anti‐infective therapy owing to their widespread availability, diverse biological activities, and relatively low toxicity. Several natural products exhibit direct antibacterial effects against common pathogenic bacteria through varied mechanisms, including disruption of cell membranes, inhibition of cell wall synthesis, or interference with nucleic acid and protein synthesis [13, 14, 15, 16]. Additionally, they may synergize with antibiotics to counteract bacterial resistance [17]. However, the poor intestinal absorption of plant‐derived natural products severely limits their clinical translation. Most natural products, characterized by complex molecular structures and poor lipid or water solubility, undergo enzymatic degradation or interact with food components in the gastrointestinal tract following oral administration, resulting in extremely low bioavailability [18]. This hinders their ability to achieve effective antimicrobial concentrations in blood and target tissues necessary for direct antibacterial action. Consequently, the mechanisms underlying the anti‐infective efficacy of natural products, particularly against non‐intestinal infections, require further investigation.

Accumulating evidence indicates that orally administered plant‐derived natural products are typically not directly absorbed by the host but rather ameliorate various metabolic disorders, including atherosclerosis [19], obesity [20], and ulcerative colitis [21], by modulating the gut microbiota [18, 22]. Gut microbiota plays a crucial role in host defense against diverse infectious agents, and its diversity is closely associated with host health status [23]. It maintains relative equilibrium by regulating homeostasis in response to external environmental factors [24, 25]. Intervening in the microbiome presents a promising approach to diminish inflammatory triggers, thereby indirectly controlling non‐intestinal infections [26, 27]. For example, Segmented filamentous bacteria reprogram alveolar macrophages to resist influenza virus infection in the lungs [28]. *Bifidobacterium animalis* suppresses non‐small cell lung cancer progression and modulates tumor immunity via indole‐3‐acetic acid [29]; and *Lactobacillus plantarum* L168 ameliorates hypoxia‐induced pulmonary inflammation and alveolar dysfunction in a rat model of bronchopulmonary dysplasia [30]. Nevertheless, the specific roles and molecular mechanisms by which natural product‐induced gut microbiota alterations protect against CRKP lung infections remain unclear.

In this study, we investigated the protective effects of plant‐derived natural products against CRKP‐induced lung infections using a mouse model and elucidated the pivotal role of the gut microbiota. Notably, we found that mice with reduced CRKP bacterial burdens exhibit elevated intestinal *Lactobacillus* levels. Mechanistically, the key probiotic *L. murinus* delivers mim‐miR‐21a‐5p to the lungs via membrane vesicles (MVs), thereby restoring mitochondrial integrity, maintaining macrophage oxidative phosphorylation, and promoting M2 polarization to protect against CRKP lung infection. Collectively, our findings highlight the therapeutic potential of plant‐derived natural products against CRKP pulmonary infection and elucidate the role of the gut‐lung axis mediated by *Lactobacillus*.

## RESULTS

### Plant‐derived natural products protect against CRKP pneumonia

To identify plant‐derived natural products capable of protecting against CRKP infections, we screened 12 clinically documented compounds, including sesamin, resveratrol, proanthocyanidins, baicalein, sanguinarine chloride, quercetin, plumbagin, luteolin, sodium houttuyfonate, umbelliferone, isochlorogenic acid A, and ginkgetin, in a mouse pulmonary infection model. Mice received daily intragastric administration of individual compounds for 5 days prior to intranasal CRKP challenge (Figure 1A,B). Post‐infection analysis revealed divergent outcomes: six compounds significantly reduced pulmonary bacterial burdens relative to vehicle‐treated controls (designated effective group, E), whereas six showed no reduction (non‐effective group, N) (Figure 1C). Histopathological analysis corroborated these findings. Specifically, lungs from group E mice retained relatively intact alveolar architecture, whereas group C exhibited mild alveolar wall thickening. In stark contrast, group N displayed severe pulmonary pathology, including hemorrhage, alveolar collapse, diminished capillary density, and structural deformities (Figure 1D–E, Figure S1A). These data suggest that specific plant‐derived natural products confer protection against CRKP‐induced lung infection.

**FIGURE 1:**
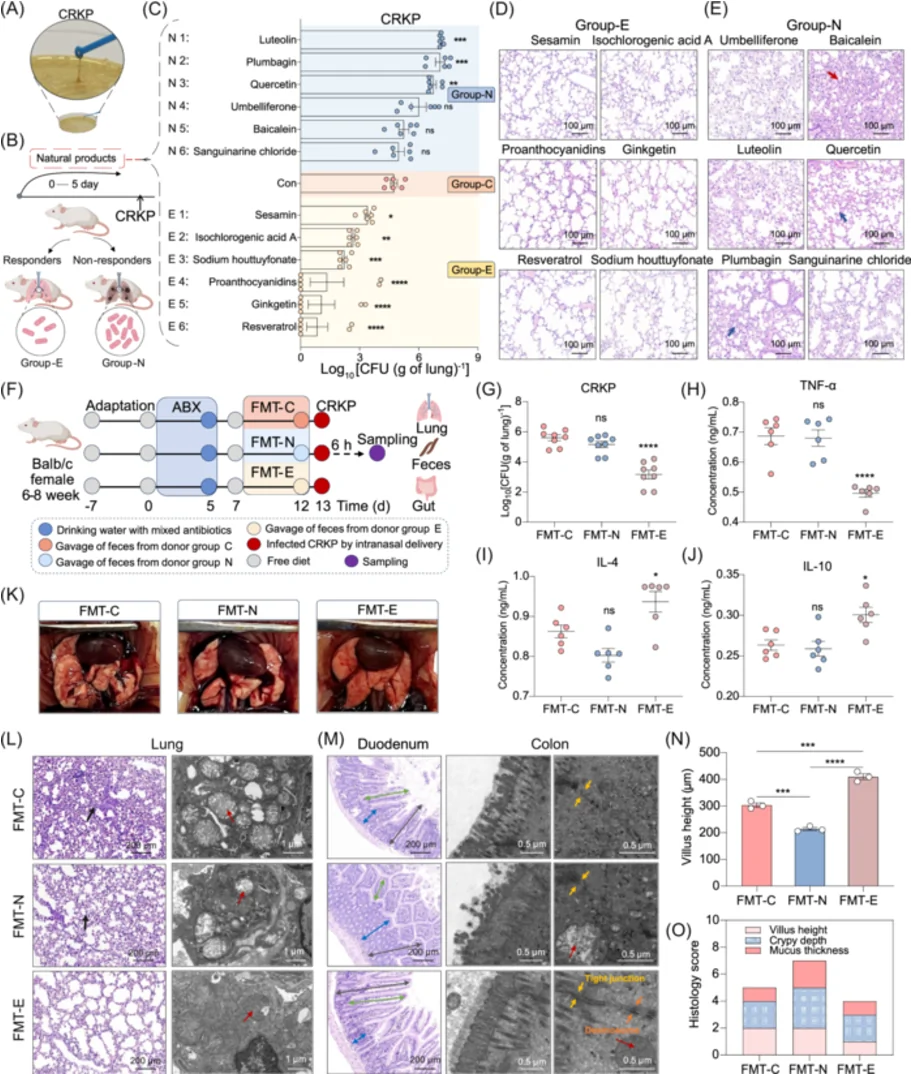
Plant‐derived natural products protect against Carbapenem‐resistant *Klebsiella pneumoniae* (CRKP) pneumonia. (A, B) Establishment of a mouse lung infection protective model (*n* = 6). Mice were pre‐administered various plant‐derived natural products for 5 days and infected with a clinical CRKP strain. Group E: effective group with significantly lower colony counts than Group C; Group N: ineffective group with higher colony counts than Group C. (C) Bacterial loads of CRKP in the lungs of mice supplemented with various natural products (*n* = 6). (D, E) Representative images of hematoxylin‐eosin (HE)‐stained sections of the lung (scale bars, 100 μm). Red arrow, alveolar collapse. Blue arrow, alveolar wall thickening. (F) Establishment of a mouse fecal microbiota transplantation (FMT) model. Antibiotics‐containing drinking water was administered for five consecutive days to deplete the gut microbiota, and free water was provided for another 2 days, followed by FMT (*n* = 8). (G) Bacterial loads of CRKP in the lungs of the FMT model (*n* = 8). (H–J) Concentrations of pro‐inflammatory (TNF‐α) and anti‐inflammatory (IL‐4 and IL‐10) factors in the FMT model (*n* = 6). (K) Clinical morphological observations of the thoracic cavity in the FMT model. (L) Representative images of HE‐stained sections and TEM of the lung in the FMT model (scale bars, 200 μm, 1 μm). Blue arrow, alveolar wall thickening. Red arrow, mitochondria. (M) Representative images of the duodenum and colon in the FMT model (scale bars, 200 μm, 0.5 μm). Green arrow, villus height. Blue arrow, crypt depth. Gray arrow, mucus thickness. Yellow arrow, tight junctions; Orange arrow, desmosome; Red arrow, mitochondria. Statistical analyses of villus height (N) and histology scores (O) (*n* = 3). Data are presented as the mean ± SEM. *n* values represent the number of biological replicates. One‐way ANOVA with Tukey's post hoc test was used to calculate *p* values in (C), (G–J), and (N). *p < 0.05, **p < 0.01, ***p < 0.001, and ****p < 0.0001. ns, not significant.

Given the limited bioavailability of these compounds, we hypothesized that gut microbiota alteration underlies their protective effects. To test this, we performed fecal microbiota transplantation (FMT) from product‐treated donor mice into naive recipients prior to CRKP challenge (Figure 1F). Consequently, FMT from effective product‐treated donors (FMT‐E) markedly reduced pulmonary CRKP loads compared to control FMT (FMT‐C) (*p* < 0.0001; Figure 1G). FMT‐E recipients also exhibited attenuated clinical disease severity (Figure S1B–D), reduced systemic and pulmonary inflammation (Figure 1H–J, Figure S1E), and absent pulmonary hemorrhage (Figure 1K, Figure S1F). Histologically, FMT‐E mice preserved alveolar integrity and mitochondrial ultrastructure, whereas FMT‐N mice showed thickened and collapsed alveolar walls with disorganized mitochondrial cristae (Figure 1L).

Intestinal barrier integrity is a prerequisite for microbiota‐mediated host protection [24]. Consistently, FMT‐N recipients displayed duodenal villus atrophy, crypt hyperplasia, and mucus layer depletion (Figure 1M,N, Figure S1G–I), alongside compromised colonic tight junctions and desmosomes (Figure 1M, Figure S1J). In contrast, FMT‐E mice maintained intact villus architecture with lower histopathological scores (Figure 1O). To rule out the interference of residual natural products in the gastrointestinal tract, we depleted the gut microbiota of product‐treated mice prior to CRKP challenge (Figure S1K). The results indicated that microbiota‐depleted mice showed no difference in pulmonary bacterial loads or inflammatory markers across treatment groups (Figure S1L–P). These findings suggest that the gut microbiota, rather than direct compound effects, may mediate the observed protection against CRKP pulmonary infection.

### Intestinal *Lactobacillus* expansion mediates resistance to CRKP infection

To characterize gut microbiota alterations induced by plant‐derived natural products, we conducted 16S rRNA sequencing analysis. α‐diversity metrics (Simpson, coverage, and Chao1 indices) and β‐diversity analyses (PCoA and NMDS) revealed distinct microbial community structures across the three experimental groups (Figure S2A–E). Taxonomic analysis identified significant differences at both phylum and genus levels (Figure 2A,B). *Lactobacillus*, *Staphylococcus*, and *norank_f_Muribaculaceae* represented the three most abundant genera, with significant intergroup variation (Figure 2C). Notably, *Lactobacillus* showed marked enrichment in group E (Figure 2D,E) and achieved the highest importance in random forest analysis (Mean Decrease Accuracy = 10.49; Figure 2F). Linear discriminant analysis with effect size measurement (LEfSe) further identified *Lactobacillus* as the most discriminative taxon (LDA score > 5; Figure 2G).

**FIGURE 2:**
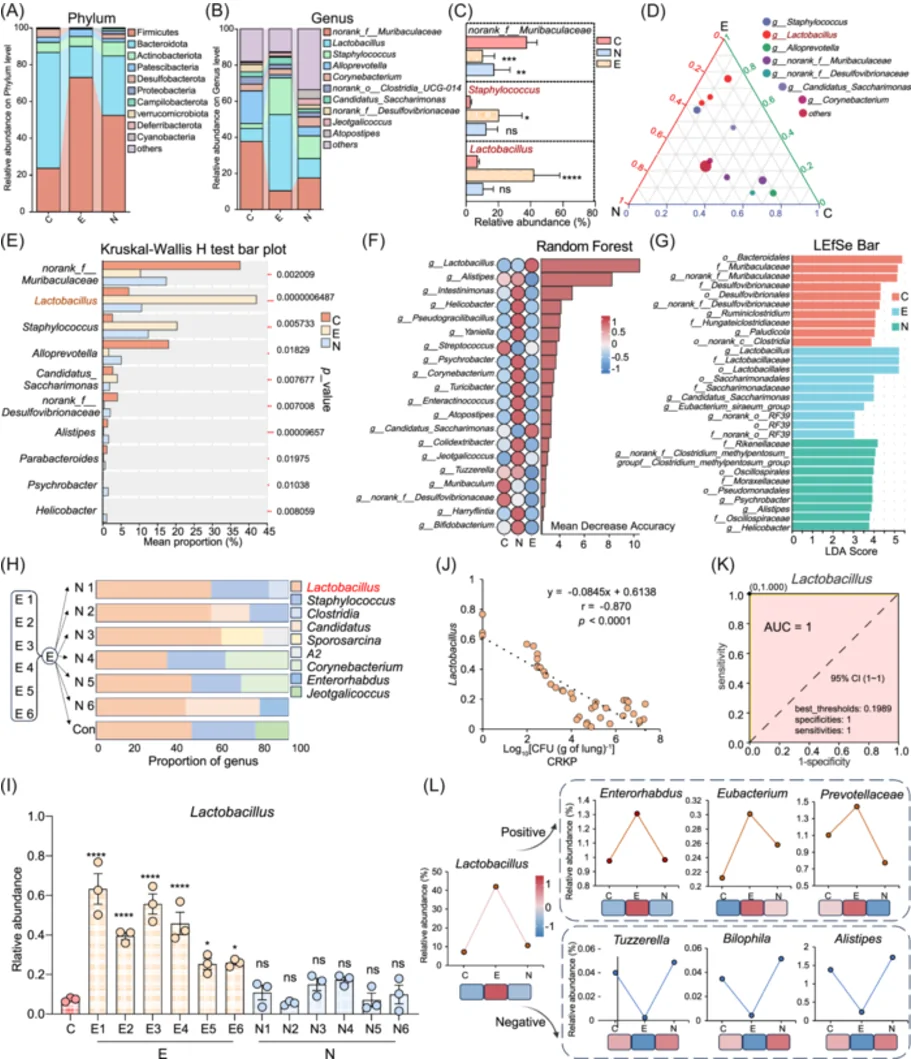
*Lactobacillus* as a dominant genus protects against CRKP infection. (A) The top 10 most abundant gut microbiota components at the phylum level. (B) The top 10 most abundant gut microbiota components at the genus level. (C) Differences in the proportion of sequences of gut microbiota components (*norank_f_Muribaculaceae*, *Staphylococcus*, and *Lactobacillus*, respectively) at the genus level (*n* = 6). (D) Ternary diagram showing the enrichment of species in the C, N, and E groups in mice. The colors in the upper right corner represent the genus level. Bubbles in the ternary diagram represent species at the genus level. Each corner of the triangle represents a group. The closer the distance from the bubble to the corners, the larger the proportion in the group. The size of the bubble indicates the species abundance. (E) Top 10 genera in abundance with significant differences among groups in mice. (F) Analysis of Random Forest at genus levels of gut microbiota. Red bubble, relatively high abundance. Blue bubble, relatively low abundance. Histogram, Mean Decrease Accuracy. (G) Linear discriminant analysis effect size from order level to genus level of gut microbiota. (H) Representative results of Figures S2F–H and S3. At the genus level, pairwise comparisons were performed between the E group (6 samples) and the N group (6 samples), and between the E group and the Con group, respectively (Related to Figures S2 and S3). The length of the bars in the bar chart indicates the proportion of subgroups accounted for by the top 3 genera with significantly increased relative abundance in Group E, compared with Group N (N1–N6) or Group Con. (I) Relative abundance of *Lactobacillus* in groups. (J) Correlation coefficients between CRKP and *Lactobacillus* in the model for natural products supplementation. (K) Receiver operating characteristic (ROC) analysis of *Lactobacillus* in the model for natural products supplementation. (L) Proportion of sequences of beneficial (*Enterorhabdus*, *Eubacterium*, and *Prevotellaceae*) and harmful bacteria (*Tuzzerella*, *Bilophila*, and *Alistipes*) and the correlation with *Lactobacillus* in mice. Red, relatively high proportion. Blue, relatively low proportion. Data in (C) and (I) are presented as the mean ± SEM. Data in (E) and (L) are presented as the mean. *n* values represent the number of biological replicates. Kruskal–Wallis test with Dunn's post hoc test was used to calculate *p* values in (C) and (E). One‐way ANOVA with Tukey's post hoc test was used to calculate *p* values in (I). *p < 0.05, **p < 0.01, ***p < 0.001, and ****p < 0.0001. ns, not significant.

Pairwise comparisons of individual natural product treatment groups confirmed that *Lactobacillus* exhibited the greatest abundance enrichment and differential expression between effective (E) and non‐effective (N) treatments (Figure 2H, Figures S2F–H, S3). The elevated *Lactobacillus* abundance in group E showed a strong negative correlation with pulmonary CRKP loads (Pearson's *r* = −0.870, *p* < 0.0001; Figure 2I,J). Receiver operating characteristic (ROC) analysis demonstrated perfect discriminatory capacity (AUC = 1.0; Figure 2K), indicating that intestinal *Lactobacillus* abundance predicts resistance to CRKP infection. Moreover, co‐occurrence network analysis revealed that *Lactobacillus* positively correlated with beneficial taxa including *Enterorhabdus*, *Eubacterium*, and *Prevotellaceae*, while negatively associating with potentially pathogenic genera such as *Tuzzerella*, *Bilophila*, and *Alistipes* (Figure 2L). These data imply that *Lactobacillus* may be an important mediator contributing to protection against CRKP infection.

### *Lactobacillus* pre‐colonization enhances lung resistance via the gut‐lung axis

To validate the protective role of *Lactobacillus* at the strain level, we isolated and characterized four distinct species from the cecal contents of group E mice: *Lactobacillus reuteri* (*L. r*), *L. taiwanensis* (*L. t*), *L. johnsonii* (*L. j*), and *L. murinus* (*L. m*) (Figure 3A,B). Mice received daily gavage of individual strains for 7 days prior to CRKP challenge (Figure 3C). Among the four strains, we found that only *L. murinus* pre‐treatment significantly reduced pulmonary CRKP burdens compared to vehicle controls (*p* < 0.0001; Figure 3D). Notably, *L. murinus* established stable intestinal colonization both before (18 h, *p* < 0.0001) and after (26 h, *p* < 0.0001) CRKP infection (Figure 3E), and maintained high abundance in FMT‐E recipients (Figure S4A).

**FIGURE 3:**
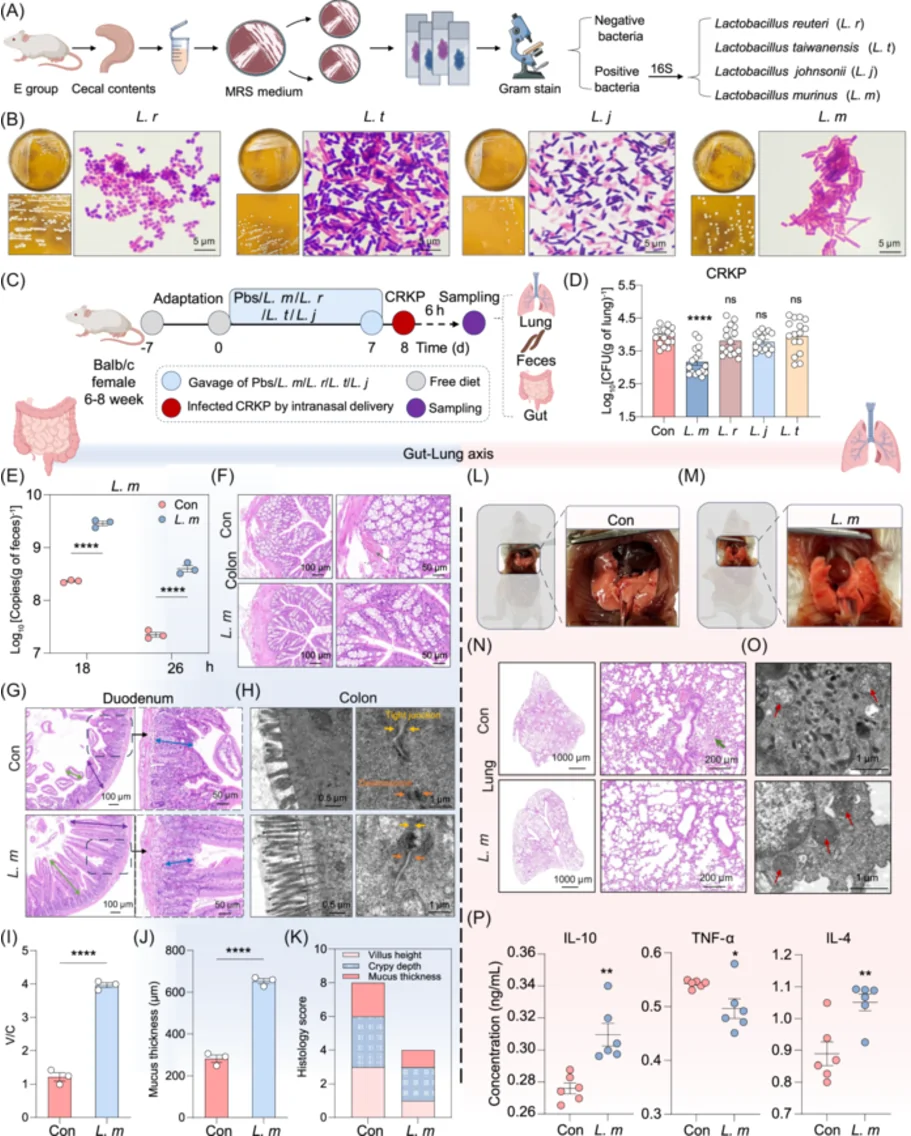
*Lactobacillus murinus* (*L. murinus*) protects against CRKP infection via the gut‐lung axis. (A) Isolation of *L. reuteri, L. taiwanensis, L. johnsonii*, and *L. murinus* from mouse cecal contents. (B) Representative images of Gram staining for *L. reuteri, L. taiwanensis, L. johnsonii*, and *L. murinus* (scale bars, 5 μm). (C) Establishment of mouse models supplemented with *L. reuteri, L. taiwanensis, L. johnsonii*, and *L. murinus* (*n* = 16). (D) Bacterial loads of CRKP in mouse lungs at 6 h post‐infection (*n* = 16). (E) Number of copies of *L. murinus* in feces before (16 h) and after (26 h) CRKP infection (*n* = 3). Representative images of HE‐stained sections of the colon (F) and duodenum (G) (scale bars, 100 μm, 50 μm). Green arrow, villus height. Blue arrow, crypt depth. Purple arrow, mucus thickness. Gray arrow, neutrophils. (H) Representative images from transmission electron microscopy (TEM) of the colon (scale bars, 0.5 μm, 1 μm). Yellow arrow, tight junctions. Orange arrow, desmosome. Statistical analyses of the V/C (I), mucus thickness (J), and histology scores (K). (*n* = 3). V/C, the ratio of villus height to crypt depth. (L, M) Clinical morphological observations of the thoracic cavity in mice. (N) Representative images of HE‐stained sections of the lung (scale bars, 100 μm, 200 μm). Green arrow, alveolar collapse. (O) Representative images of TEM of the lung (scale bars, 1 μm). Red arrow, mitochondria. (P) Concentrations of pro‐inflammatory (TNF‐α) and anti‐inflammatory (IL‐10 and IL‐4) factors in mice (*n* = 6). Data are presented as the mean ± SEM. *n* values represent the number of biological replicates. Kruskal–Wallis test with Dunn's post hoc test was used to calculate *p* values in (D). Unpaired two‐tailed Student's *t* test was used to calculate *p* values in (E), (I, J), and (P). *p < 0.05, **p < 0.01, ***p < 0.001, and ****p < 0.0001. ns, not significant.

*L. murinus* is a predominant commensal bacterium critical for intestinal epithelial integrity and barrier function [31]. We therefore examined its effects on gut architecture. Consequently, *L. murinus* supplementation ameliorated colonic goblet cell hypertrophy (Figure 3F), preserved duodenal villus structure, and enhanced tight junction and desmosome integrity in colonic epithelium (Figure 3G–J, Figure S4B), resulting in reduced histological scores (Figure 3K).

Given that gut homeostasis is intimately linked to pulmonary health through inter‐organ crosstalk, we further assessed lung outcomes. Notably, we found that *L. murinus* pre‐treatment attenuated clinical disease severity (Figure 3L,M, Figure S4C,D), prevented alveolar wall thickening and collapse (Figure 3N), restored mitochondrial cristae integrity (Figure 3O), reduced apoptotic cell infiltration (Figure S4E), and dampened pulmonary inflammation (Figure 3P, Figure S4F). These findings suggest that intestinal colonization with *L. murinus* enhances lung resistance to CRKP infection through the gut–lung axis.

### *L. murinus*‐derived sRNA and MVs protect against CRKP infection

To dissect the mechanism underlying *L. murinus*‐mediated protection, we first tested cell‐free supernatant (*L. m*‐SNF) containing metabolites such as short‐chain fatty acids and bacteriocins. However, *L. m*‐SNF failed to reduce pulmonary CRKP burdens (Figure 4A), suggesting that unstable or dilute factors were insufficient for protection. We next heat‐inactivated *L. murinus* at 85°C (HK‐*L. m*) to facilitate the release of stable intracellular components. The results showed that HK‐*L. m* significantly reduced lung bacterial loads compared to controls (*p* = 0.0015; Figure 4A). This protective effect was recapitulated by HK‐*L. m* supernatant (HK‐*L. m* ^SNF^) but not by heat‐killed bacterial cells (HK‐*L. m* ^Cells^) (Figure 4B), indicating that stable secreted factors mediate protection. Ultrasonic disruption (UL‐*L. m*) confirmed that physical cell lysis accelerates factor release. Electron microscopy revealed that heat treatment preserved bacterial ultrastructure while inducing surface protrusions consistent with content release upon cooling (Figure 4C). To assess whether this mechanism extends to other *Lactobacillus* species, we tested heat‐inactivated *L. reuteri*, *L. taiwanensis*, and *L. johnsonii* (HK‐*L. r*, HK‐*L. t*, HK‐*L. j*). All three strains reduced pulmonary CRKP loads to varying degrees (Figure S5A), indicating that heat‐stable factors are conserved across protective *Lactobacillus* species, with *L. murinus* exhibiting the most potent effect.

**FIGURE 4:**
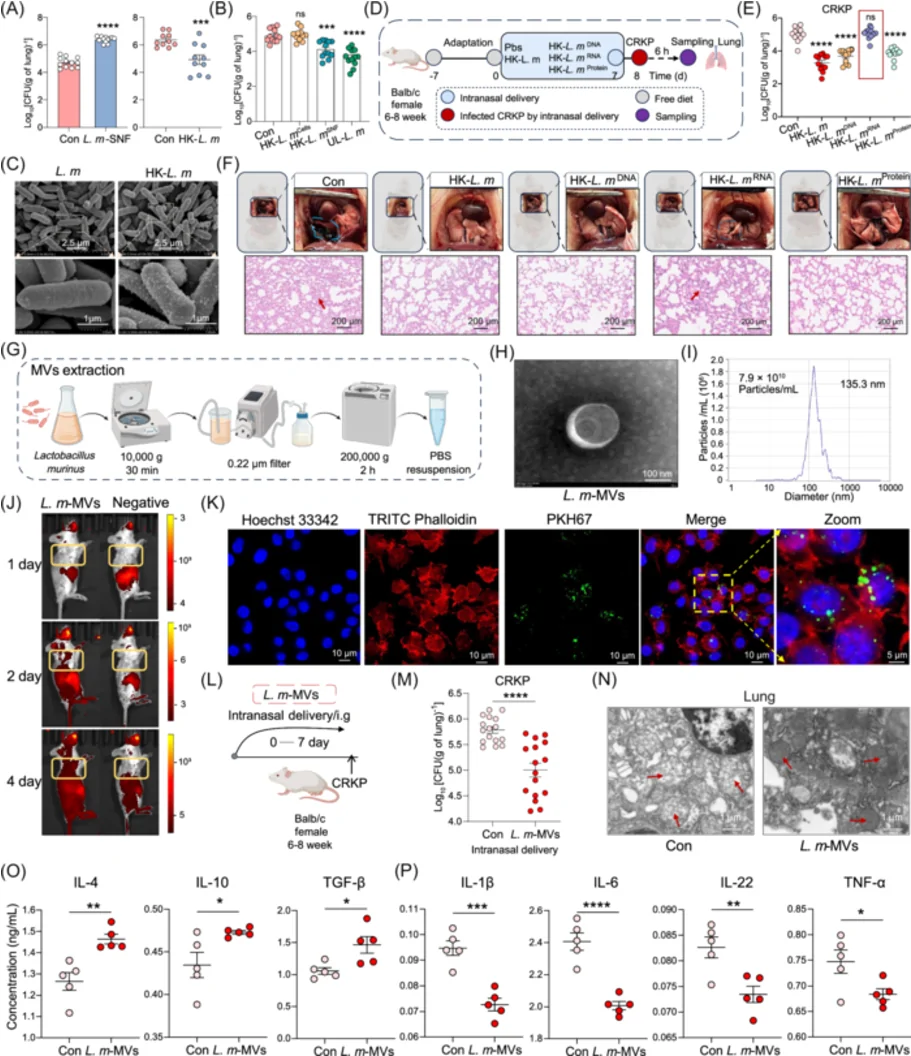
*L. murinus*‐derived membrane vesicles (MVs) and sRNAs confer protection against CRKP pneumonia. (A) Bacterial loads of CRKP in mouse lungs after supplementation with *L. m*‐SNF (*n* = 12) and HK‐*L. m* (*n* = 10). (B) Bacterial loads of CRKP in mouse lungs after supplementation with HK‐*L. m* ^Cells^, HK‐*L. m* ^SNF^, and UL‐*L. m* (*n* = 12). (C) Representative images from a scanning electron microscope (SEM) of *L. m* and HK‐*L. m* (scale bars, 2.5 μm, 1 μm). (D) Establishment of a mouse model supplemented with HK‐*L. m* digested by DNase I, RNase A, and Proteinase K (*n* = 12). (E) Bacterial loads of CRKP in mouse lungs of (D). (F) Clinical morphological observations of the thoracic cavity and representative images of lung HE‐stained sections in model (D). (Scale bars, 200 μm). Red arrow, alveolar collapse. (G) Extraction of MVs by ultracentrifugation. (H) Representative TEM images of MVs (scale bars, 100 nm). (I) The nanoparticle tracking analysis (NTA) of MVs. (J) Representative images of live imaging of MVs uptake in mice. DiR‐labeled MVs were resuspended in sterile PBS, and mice were intragastrically administered 400 μg of MVs in 300 μL PBS, with an equal amount of DiR dye serving as the negative control. (K) Representative images of immunofluorescence of macrophages internalizing MVs (scale bars, 10 μm, 5 μm). (L) Establishment of a mouse model with *L. m*‐MVs supplementation (*n* = 16). (M) Bacterial load of CRKP in mouse lungs in (L). (N) Representative images of lung TEM in model (L) (scale bars, 1 μm). Red arrow: mitochondria. (O, P) Concentrations of anti‐inflammatory (IL‐4, IL‐10, and TGF‐β) and pro‐inflammatory (TNF‐α, IL‐22, IL‐1β, and IL‐6) factors in mouse lungs (*n* = 5). SNF, supernatant; HK, heat‐killed; UL, ultrasonic disruption. Data are presented as the mean ± SEM. *n* values represent the number of biological replicates. Unpaired two‐tailed Student's *t* test was used to calculate *p* values in (A), (M), and (O, P). One‐way ANOVA with Tukey's post hoc test was used to calculate *p* values in (B) and (E). *p < 0.05, **p < 0.01, ***p < 0.001, and ****p < 0.0001. ns, not significant.

To investigate the bioactive components derived from *L. murinus* that protect against CRKP infection, we pretreated HK‐*L. m* with DNase I, RNase A, and proteinase K, respectively prior to administration (Figure 4D). Only RNase A abrogated protection, as evidenced by unchanged bacterial loads (Figure 4E), persistent clinical symptoms (Figure 4F), and unabated inflammation (Figure S5B–D). RNase A sensitivity was also confirmed with UL‐*L. m* (Figure S5E,F). Additionally, biochemical analysis showed minimal protein in HK‐*L. m* ^SNF^, whereas RNA and DNA concentrations were elevated, with RNA levels comparable to UL‐*L. m* (Figure S5G–I). These data identify RNA as the critical protective component derived from *L. murinus*.

Given the susceptibility of naked RNA to degradation, we hypothesized that *L. murinus* may package protective RNA into membrane vesicles (MVs) for delivery to the lungs. To test this hypothesis, we extracted *L. murinus*‐derived MVs through ultracentrifugation (Figure 4G), which exhibited characteristic cup‐shaped morphology (Figure 4H) and diameters of 20–300 nm (Figure 4I). Live imaging results indicated that DIR‐labeled MVs were detected in lungs 48 h after administration (Figure 4J). In vitro experiments demonstrated that PKH67‐labeled MVs were internalized by macrophages after 8 h of co‐incubation (Figure 4K, Figure S5J). Most importantly, mice receiving MVs for 7 days prior to CRKP challenge showed markedly reduced pulmonary bacterial loads, preserved alveolar architecture (*p* < 0.0001; Figure 4L,M, Figure S5K,L), intact mitochondrial ultrastructure (Figure 4N), and attenuated inflammation (Figure 4O,P). These findings suggest that MVs derived from *L. murinus* confer protection against CRKP infection.

### MVs‐encapsulated mim‐miR‐21a‐5p alleviates CRKP pneumonia

To verify whether sRNAs are encapsulated within MVs and identify the biologically active RNA species mediating the protective effect, we performed small RNA sequencing on lung tissues from HK‐*L. m*‐treated mice, as well as on HK‐*L. m* and derived MVs (Figure 5A). Using miRBase annotation, we identified bacterial sRNAs with sequence homology to mouse microRNAs (designated mim‐miRNAs). The HK‐*L. m* group exhibited 250 known sRNAs in lung tissue compared to 244 in controls, while MVs and HK‐*L. m* contained 104 and 107 known sRNAs, respectively (Figure 5B). Venn analysis of upregulated sRNAs in lung tissue, bacterial‐derived sRNAs, and stably expressed MVs‐sRNAs revealed seven common candidates: mim‐let‐7f‐5p, mim‐miR‐152, mim‐miR‐16, mim‐miR‐29c, mim‐miR‐22‐3p, mim‐miR‐21a‐5p, and mim‐miR‐100 (Figure 5C,D). All seven were detected in *L. m*‐MVs (Figure 5E) and showed high expression in both MVs and HK‐*L. m* (Figure S6A,B). Moreover, following HK‐*L. m* supernatant treatment, these sRNAs were upregulated in lung tissue to varying degrees (Figure S6C,D).

**FIGURE 5:**
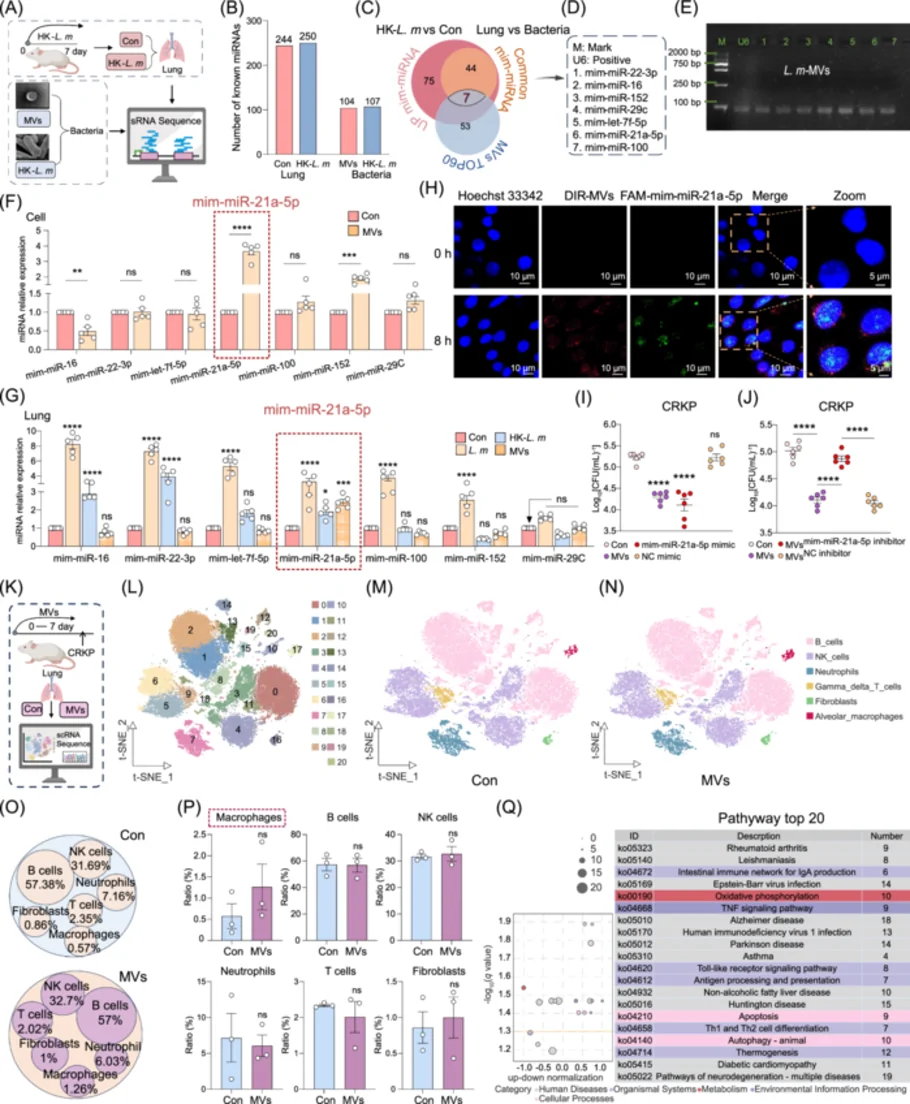
MVs‐delivered mim‐miR‐21a‐5p sustains macrophage oxidative phosphorylation (OXPHOS). (A) Schematic diagram of sRNA Sequencing (*n* = 3). (B) Number of known sRNAs in each group. (C) Venn analysis of the upregulated sRNAs in the HK‐*L. m* group of lung tissues, the sRNAs shared between lung tissues and bacteria, and the stably expressed sRNAs in MVs. (D and E) Gel electrophoresis of PCR amplification products for seven sRNAs in MVs. (F) Relative expression of sRNAs in macrophages treated with MVs (*n* = 5). (G) Relative expression of sRNAs in mouse lungs treated with *L. m*, HK‐*L. m*, and MVs (*n* = 5). (H) Representative images of immunofluorescence of macrophages internalizing MVs loaded with mim‐miR‐21a‐5p (scale bars, 10 μm, 5 μm). (I) Bacterial load of CRKP in macrophages transfected with mim‐miR‐21a‐5p mimic (*n* = 6). (J) Bacterial load of CRKP in macrophages after co‐incubation with MVs loaded with mim‐miR‐21a‐5p inhibitor (*n* = 6). (K) Schematic diagram of scRNA Sequencing (*n* = 3). (L) t‐SNE distributions of 20 clusters identified and classified in lung cells. t‐SNE, t‐distributed stochastic neighbor embedding. (M, N) t‐SNE distribution of 20 clusters grouped into 6 cell clusters in the Con group and MVs group. (O) Schematic diagram of the relative expression ratios of six cell clusters in the Con and MVs groups. (P) Relative expression ratios of six cell clusters in the Con and MVs groups (*n* = 3). (Q) KEGG analysis of significantly DEGs in macrophages between the Con and MVs groups. KEGG, Kyoto Encyclopedia of Genes and Genomes; DEGs, differentially expressed genes. Data are presented as the mean ± SEM. *n* values represent the number of biological replicates. Unpaired two‐tailed Student's *t* test was used to calculate *p* values in (F). One‐way ANOVA with Tukey's post hoc test was used to calculate *p* values in (G), (I), and (J). Mann–Whitney *U* test was used to calculate *p* values in (P). *p < 0.05, **p < 0.01, ***p < 0.001, and ****p < 0.0001. ns, not significant.

Quantitative analysis revealed that mim‐miR‐21a‐5p exhibited the most significant increase following MVs co‐incubation with RAW264.7 cells (*p* = 0.0032; Figure 5F). In vivo, mim‐miR‐21a‐5p levels were consistently elevated in lungs following treatment with *L. m* (*p* < 0.0001), HK‐*L. m* (*p* = 0.0735), and MVs (*p* = 0.0432) compared to controls (Figure 5G), identifying mim‐miR‐21a‐5p as a candidate effector molecule. We next quantified lung‐recruited MVs and their encapsulated mim‐miR‐21a‐5p (Figure S6E). Both MVs copy numbers and particle counts increased significantly by Day 7 of *L. murinus* gavage compared with Days 3 and 5 (Figure S6F). Digital PCR revealed that mim‐miR‐21a‐5p concentrations within MVs ranged from 170 to 191 copies/μL (Figure S6G,H), and absolute copy numbers were substantially higher at Day 7 than at Days 3 and 5 (Figure S6I). Together, these findings demonstrate that MVs and encapsulated mimic miR‐21a‐5p accumulate in the lung over time, suggesting a functional role in mediating biological processes.

To verify mim‐miR‐21a‐5p as the functional component, we first loaded FAM‐labeled mim‐miR‐21a‐5p into MVs via sonication (Figure S6J) and confirmed efficient uptake by RAW264.7 cells after 8 h of incubation (Figure 5H, Figure S6K). Importantly, overexpression of mim‐miR‐21a‐5p in RAW264.7 cells significantly reduced intracellular CRKP burdens (*p* < 0.0001; Figure 5I) with concomitant increase in mim‐miR‐21a‐5p expression (Figure S6L). Conversely, MVs loaded with mim‐miR‐21a‐5p inhibitors lost their protective capacity, resulting in increased bacterial loads (*p* < 0.0001; Figure 5J) and reduced mim‐miR‐21a‐5p levels (Figure S6M). These results suggest mim‐miR‐21a‐5p as the key effector RNA within *L. m*‐MVs that mediates resistance to CRKP infection. We further examined MVs from *L. reuteri, L. taiwanensis*, and *L. johnsonii*. All contained mim‐miR‐21a‐5p, with *L. m*‐MVs showing the highest abundance (Figure S6N,O), consistent with the superior protective efficacy of *L. murinus* and supporting its dominant role within the protective *Lactobacillus* community.

### MVs sustain oxidative phosphorylation homeostasis in macrophages

We have observed that *L. m*, HK‐*L. m*, and MVs confer protection against CRKP infection in a mouse model of acute CRKP infection. To assess the therapeutic potential of *L. murinus* and derived products in a persistent infection context, we established a chronic CRKP infection model involving serial challenges every 48 h for 2 weeks, followed by treatment with *L. m*, HK‐*L. m*, or MVs (Figure S7A). Notably, all three treatments significantly reduced bacterial burdens in lungs (*p* < 0.0001, *p* < 0.0001, and *p* = 0.0263), liver (*p* = 0.0003, *p* = 0.0002, and *p* = 0.0146), and kidneys (*p* = 0.0012, *p* = 0.0048, and *p* < 0.0001) compared to controls (Figure S7B,C).

To determine whether protection occurs through direct antibacterial activity, we performed in vitro assays. The results showed that co‐culture of *L. m* with CRKP in aerobic LB or anaerobic MRS medium did not inhibit bacterial growth (Figure S7D,E). The minimum inhibitory concentration (MIC) of both HK‐*L. m* and MVs against CRKP exceeded 256 μg/mL (Figure S7F), and MVs supplementation did not alter CRKP growth kinetics (Figure S7G). These findings exclude direct antimicrobial effects and indicate that protection is mediated through host‐directed mechanisms.

To identify the cellular targets of MVs, we performed single‐cell RNA sequencing (scRNA‐seq) on lung tissue from MVs‐treated mice (Figure 5K). Clustering analysis identified 20 transcriptionally distinct populations, annotated as B cells, NK cells, neutrophils, γδ T cells, fibroblasts, and alveolar macrophages (Figure 5L–N). Notably, MVs treatment increased macrophage proportions by 2.2‐fold compared to controls (Figure 5O,P). Differential expression analysis of macrophages revealed 237 upregulated and 164 downregulated genes (Figure S7H). Gene Ontology (GO) analysis indicated enrichment in intracellular structural organization and metabolic processes (Figure S7I), concordant with sRNA sequencing GO results (Figure S7J). Kyoto Encyclopedia of Genes and Genomes (KEGG) pathway analysis further identified oxidative phosphorylation (OXPHOS) as the most significantly enriched metabolic pathway (Figure 5Q). Given the established mitochondrial protective effects of *L. m* and MVs (Figures 1, 3, 4) and the central role of mitochondria in OXPHOS, we hypothesized that MVs‐delivered mim‐miR‐21a‐5p restores macrophage mitochondrial integrity and metabolic homeostasis to enhance CRKP resistance.

### mim‐miR‐21a‐5p targets *PDCD4* to activate Nrf2/SOD2 and maintain OXPHOS

To identify the mechanism by which mim‐miR‐21a‐5p modulates macrophage metabolism, we predicted target genes using miRWalk and TargetScan. Screening for candidates linking macrophage function and OXPHOS identified *PDCD4* and *PTEN* (Figure S8A). Among these, only *PDCD4* showed consistent downregulation in both in vivo (*L. m*, *p* = 0.0002; HK‐*L. m*, *p* < 0.0001; MVs, *p* < 0.0001; Figure 6A) and in vitro (*p* = 0.0002; Figure S8B) experiments. Luciferase reporter assays confirmed direct targeting of *PDCD4* by mim‐miR‐21a‐5p (Figure 6B). To further strengthen the regulatory link and mechanism between mim‐miR‑21a‑5p and *PDCD4*, we examined the mRNA and protein levels of PDCD4 in gain‑ and loss‑of‑function assays of mim‐miR‑21a‑5p. The results showed that mim‐miR‐21a‐5p overexpression reduced PDCD4 mRNA and protein levels (Figure 6C,D), whereas inhibition increased PDCD4 expression (Figure S8C,D). These results suggest *PDCD4* as a potential target gene of mim‐miR‑21a‑5p.

**FIGURE 6:**
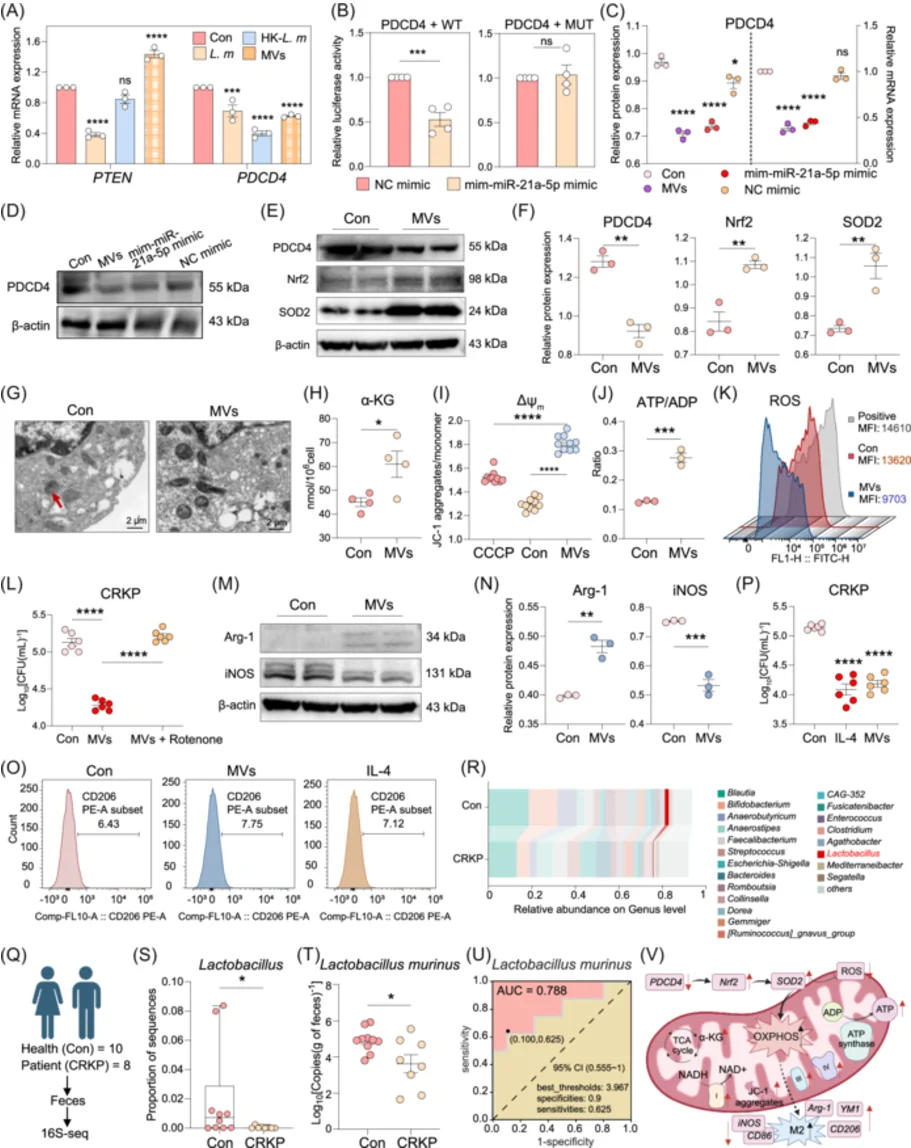
*PDCD4* suppression activates Nrf2/SOD2 to sustain OXPHOS and M2 polarization. (A) mRNA relative expression of *PTEN* and *PDCD4* in mouse lungs treated with *L. m*, HK‐*L. m*, and MVs (*n* = 3). (B) Luciferase activities in macrophages co‐transfected with luciferase reporter plasmid plus mim‐miR‐21a‐5p or NC mimics (*n* = 4). WT: Wild‐Type plasmid. MUT: Mutant plasmid. (C, D) PDCD4 mRNA and protein levels in macrophages transfected with mim‐miR‐21a‐5p mimic (*n* = 3). (E, F) Western blots showing PDCD4, Nrf2, and SOD2 expression in macrophages treated with MVs (*n* = 3). (G) Representative TEM images of MVs‐treated macrophages. Red arrow: mitochondria. (H) Concentrations of α‐KG in macrophages treated with MVs (*n* = 4). α‐KG, α‐Ketoglutaric acid. (I) The ΔΨm changes in macrophages treated with MVs (*n* = 10). ΔΨm, JC‐1 aggregates/monomer. CCCP, Positive. (J) The ratio of ATP/ADP in macrophages treated with MVs (*n* = 3). (K) The level of ROS, measured by peak analysis in flow cytometry. Positive, Rosup. ROS, reactive oxygen species. MFI, mean fluorescence intensity. (L) Bacterial load of CRKP in macrophages treated with MVs and rotenone (*n* = 6). (M and N) Western blots showing Arg‐1 and iNOS expression in macrophages treated with MVs (*n* = 3). (O) The level of CD206 in macrophages treated with MVs, measured by flow cytometry. MFI, mean fluorescence intensity. IL‐4, Positive. (P) Bacterial load of CRKP in macrophages treated with MVs or IL‐4 (*n* = 6). (Q) Scheme representing the sample collection of healthy (*n* = 10) and CRKP patients (*n* = 8). (R) The top 22 most abundant gut microbiota components at the genus level in fecal samples from model (Q). (S) Sequence proportion of *Lactobacillus* in fecal samples from model (Q). (T) Number of copies of *Lactobacillus murinus* in fecal samples from model (Q). (U) ROC analysis of *Lactobacillus murinus* in (T). (V) Schematic diagram of *PDCD4* suppression protecting oxidative phosphorylation via the Nrf2/SOD2 pathway to maintain macrophage M2 polarization. Data are presented as the mean ± SEM. *n* values represent the number of biological replicates. Unpaired two‐tailed Student's *t* test was used to calculate p values in (B), (F), (H), (J), and (N). One‐way ANOVA with Tukey's post hoc test was used to calculate *p* values in (A), (C), (I), (L), and (O). Mann–Whitney *U* test was used to calculate *p* values in (S) and (T). *p < 0.05, **p < 0.01, ***p < 0.001, and ****p < 0.0001. ns, not significant.

*PDCD4* regulates cell proliferation, apoptosis, and oxidative stress responses [32]. It has been shown that *PDCD4* inhibition indirectly stabilizes Nrf2, a master regulator of antioxidant defense, thereby inducing superoxide dismutase 2 (SOD2) expression to preserve mitochondrial integrity and redox homeostasis [33]. Consistent with this mechanism, our results showed that MVs treatment reduced PDCD4 protein (*p* = 0.0013) while increasing Nrf2 and SOD2 expression at both protein (*p* = 0.0054 and *p* = 0.0086) and mRNA (*p* = 0.0033 and *p* < 0.0001) levels (Figure 6E,F, Figure S8E). To directly verify the activating and protective effects of *PDCD4* inhibition on the Nrf2/SOD2 pathway, we knocked out *PDCD4* in RAW264.7 cells. The results showed that genetic deletion of *PDCD4* in RAW264.7 cells phenocopied these effects, reducing *PDCD4* mRNA (*p* < 0.0001), elevating *Nrf2* and *SOD2* expression (*p* = 0.0102 and *p* = 0.0005), and decreasing intracellular CRKP burdens (*p* < 0.0001; Figure S8F–H). scRNA‐seq analysis further revealed that macrophages (cluster 17) exhibited the lowest *PDCD4* and highest *SOD2* expression among all lung cell populations (Figure S8I,J). These data suggest that mim‐miR‐21a‐5p‐mediated *PDCD4* suppression activates the Nrf2/SOD2 antioxidant axis to enhance CRKP resistance.

We next assessed mitochondrial status and oxidative phosphorylation. Transmission electron microscopy revealed orderly cristae and preserved ultrastructure in MVs‐treated RAW264.7 cells (Figure 6G). Moreover, MVs treatment elevated tricarboxylic acid (TCA) cycle activity, as evidenced by increased α‐ketoglutarate levels (*p* = 0.034; Figure 6H), and enhanced electron transport chain Complex III/IV activities and succinate dehydrogenase (*p* < 0.0001, *p* = 0.0223, and *p* = 0.0053; Figure S8K,L). Meanwhile, functional assays confirmed that MVs treatment restored mitochondrial membrane potential (*p* < 0.0001; Figure 6I, Figure S8M), increased ATP/ADP ratio (*p* = 0.0007; Figure 6J, Figure S8N), and reduced reactive oxygen species (*p* < 0.0001; Figure 6K, Figure S8O). Critically, the addition of the oxidative phosphorylation inhibitor rotenone abolished MVs‐mediated bacterial clearance (*p* < 0.0001; Figure 6L), indicating OXPHOS restoration as the essential mechanism of protection.

### OXPHOS promotes macrophage M2 polarization against CRKP infection

OXPHOS sustains the bioenergetic demands of alternatively activated (M2) macrophages [34]. Our results indicated that MVs treatment significantly elevated arginase‐1 (Arg‐1) protein (*p* = 0.0014) while reducing inducible nitric oxide synthase (iNOS) protein (*p* = 0.0004; Figure 6M,N), indicating a shift from pro‐inflammatory M1 toward anti‐inflammatory M2 polarization. Concordantly, M2‐associated transcripts (*YM1, CD206, Arg‐1*, and *IL‐10*) were upregulated (*p *= 0.0005, *p* = 0.0078, *p* < 0.0001, and *p* = 0.0677; Figure S9A), whereas the M1 marker *TNF‐α* was downregulated (*p* = 0.0006; Figure S9B). Flow cytometry confirmed increased CD206 and decreased CD86 mean fluorescence intensity (Figure 6O, Figure S9C).

To establish a causal link between OXPHOS and M2 polarization, we employed rotenone. The OXPHOS inhibitor reversed MVs‐mediated reductions in ROS (*p* < 0.0001; Figure S9D) and restored mitochondrial membrane potential (*p* < 0.0001; Figure S9E,F). Functionally, rotenone blocked MVs‐induced Arg‐1 upregulation and iNOS downregulation (*p* < 0.0001; Figure S9G,H), as well as transcriptional changes in *IL‐10, Arg‐1, YM1*, and *TNF‐α* (*p* = 0.0009, *p* = 0.0003, *p* = 0.0002, and *p* = 0.0149; Figure S9I). Importantly, MVs significantly reduced intracellular CRKP burdens (*p* < 0.0001; Figure 6P), whereas rotenone abolished this effect (*p* < 0.0001; Figure 6L), demonstrating that OXPHOS‐dependent M2 polarization is essential for CRKP clearance. Consistently, scRNA‐seq analysis confirmed that macrophages (cluster 17) exhibited the highest M2 signature expression, with MVs treatment increasing the M2 macrophage proportion (Figure S9J–L).

To translate these findings into clinical implications, we performed 16S rRNA sequencing on fecal samples from healthy controls and CRKP‐infected patients (Figure 6Q). The results showed that CRKP patients exhibited altered microbial community structure (Figure 6R) and significantly reduced *Lactobacillus* abundance (*p* = 0.044; Figure 6S). Absolute quantification revealed specifically decreased *L. murinus* levels (*p* = 0.0434; Figure 6T), with an AUC of 0.788 for discriminating CRKP infection (Figure 6U). These data point to an association between CRKP infection and depletion of intestinal *Lactobacillus*, particularly reduced abundance of *L. murinus*, warranting further investigation of its potential biomarker utility. We further verified whether this protective effect extends to other clinical CRKP strains. The results showed that *Lactobacillus* and its‑derived MVs effectively reduced the pulmonary bacterial burden of CRKP isolates belonging to ST11/KL25 (C10), ST11/KL64 (C14), and ST11/KL47 (C18) (Figure S9M–O).

Collectively, our findings establish a gut–lung axis wherein *L. murinus*‐derived MVs deliver mim‐miR‐21a‐5p to pulmonary macrophages, suppressing *PDCD4* to activate the Nrf2/SOD2 antioxidant pathway. This cascade restores mitochondrial OXPHOS, sustains M2 polarization, and enhances host resistance to CRKP infection (Figure 6V, Figure S9P).

## DISCUSSION

Plant‐derived natural products have long been recognized for their potential antimicrobial properties [13, 14], yet their clinical application against multidrug‐resistant pathogens such as CRKP remains limited. In this study, we revealed that six structurally diverse natural products, including flavonoids, alkaloids, organic acid derivatives, and coumarins, conferred protection against CRKP pulmonary infection not through direct antibacterial activity, but by reshaping the gut microbiota. This aligns with emerging evidence that natural products exert systemic effects through microbiota modulation rather than isolated pharmacological targets [18, 24].

Our findings identify *Lactobacillus* as the keystone genus mediating protection, with *L. murinus* playing a dominant role. This is consistent with prior reports that certain natural products stimulate intestinal mucus secretion, thereby providing nutrients for *Lactobacillus* growth [35], and protect gut microbiota diversity through multiple pathways, including upregulation of tight junction proteins [36], modulation of intestinal immune cell function [37], and enhancement of antimicrobial peptide levels [38]. However, the regulation of natural products on intestinal microorganisms is not a simple one‐way stimulus, but a multidimensional complex system [18]. Beyond *Lactobacillus*, we observed concomitant enrichment of *Enterorhabdus, Eubacterium*, and *Prevotellaceae*, beneficial genera known to produce short‐chain fatty acids, reinforce intestinal barrier function, and regulate immune homeostasis [39, 40]. Conversely, effective natural products diminished *Tuzzerella*, *Bilophila*, and *Alistipes*, pathogenic bacteria that generate cytotoxic metabolites such as 4‐chlorophenol and hydrogen sulfide, impair mitochondrial function [41, 42, 43], and have been associated with fatty liver disease [44], colitis [45], and pancreatitis [46].

Notably, live *L. murinus* and its heat‐inactivated products were equally protective, challenging the conventional emphasis on viable probiotics. Heat‐inactivated probiotics, which accelerate bacterial metabolism without disrupting their physical structure, have been shown to mildly regulate immune function to prevent overactivation and enhance immune homeostasis, thereby alleviating obesity [47, 48], mitigating colonic aging [49], and resisting *Salmonella* infection [50]. Our findings extend this concept to CRKP resistance, revealing that heat inactivation accelerates the release of mim‐miR‐21a‐5p from multiple *Lactobacillus* species. The conservation of this mechanism across species suggests that heat‐stable, RNA‐rich preparations may represent a scalable anti‐infective strategy.

MVs are nanoscale structures with low toxicity and biocompatibility that mediate host–microbe communication by delivering bacterial signaling molecules into host cells to regulate gene expression and function [31, 51]. Our data indicated that *L. murinus*‐derived MVs translocate from the intestine to the lung, delivering mim‐miR‐21a‐5p to pulmonary macrophages. The expression of mim‐miR‐21a‐5p in *L. murinus* MVs was highest among the four species examined, highlighting *L. murinus* as a primary probiotic in CRKP resistance. This bacterial small RNA suppresses *PDCD4*, thereby derepressing the Nrf2/SOD2 antioxidant axis to restore mitochondrial OXPHOS. *PDCD4* inhibition indirectly upregulates Nrf2, a critical regulator of cellular antioxidant stress responses, thereby activating SOD2 to scavenge excess ROS [52, 53], prevent mitochondrial damage, and maintain redox balance [33]. The highly folded inner mitochondrial membrane is crucial for OXPHOS [54], which M2 macrophages depend on for energy production and anti‐inflammatory functions. The regulatory interplay among mitochondrial homeostasis, OXPHOS, and M2 macrophage polarization is critical in various inflammation‐related diseases, such as atherosclerotic cardiovascular disease [55], Alzheimer's disease [34], inflammatory bowel disease [56], and tumor immune regulation [57].

Our mechanistic insights contrast with the pathogenic M2‐like phenotype [M(Kp)] induced by *K. pneumoniae* itself, which promotes intracellular bacterial persistence through a “Trojan horse” strategy [58]. MVs pretreatment instead primes a protective M2 state that blocks bacterial immune escape at the early infection stage. This distinction underscores the context‐dependent nature of macrophage polarization: while *K. pneumoniae* drives macrophages toward pathogenic M(Kp), facilitating intracellular bacterial escape, survival, and replication, MVs‐induced metabolic reprogramming enhances resistance to infection. Given the complex multi‐stage interactions between macrophages and *K. pneumoniae*, their precise molecular mechanisms require further investigation.

Translational relevance is supported by our clinical observation that CRKP‐infected patients exhibited depleted intestinal *Lactobacillus*, particularly *L. murinus*, with moderate diagnostic discriminatory capacity. While causality remains to be established in human trials, these data suggest that *L. murinus* abundance may serve as a biomarker for infection susceptibility.

### Limitations of the study

Although this study provides new mechanistic insights into how plant‐derived natural products protect against CRKP‐induced lung infections, several limitations should be acknowledged. First, although RAW264.7 cells share key functional properties with primary alveolar macrophages, they may not fully recapitulate the complexity of their *in vivo* counterparts. Second, although oral MVs demonstrate cross‐organ efficacy, translational optimization of encapsulation efficiency, stability, and human‐equivalent dosing for cross‐kingdom RNA therapeutics remains to be established. Third, quantitative comparison of MVs production between *in vitro* and *in vivo* conditions remains technically challenging with current methodologies, requiring further refinement. Fourth, the use of several CRKP strains limits the generalizability of these findings, and validation across multiple clinically representative isolates is warranted.

## CONCLUSION

Our study establishes a mechanistic framework wherein plant‐derived natural products enhance CRKP resistance through a gut–lung–macrophage axis orchestrated by *L. murinus*‐derived MVs and their cargo, mim‐miR‐21a‐5p. This work advances the concept of microbiota‐directed therapies against antimicrobial‐resistant pathogens and supports the development of heat‐stable, RNA‐based biologics for infection prevention.

## METHODS

### Mice

Specific‐pathogen‐free‐grade Balb/c female mice were procured from the Institute of Comparative Medicine at Yangzhou University. In all experiments, female mice aged 6 to 8 weeks were utilized. The mice were housed within a barrier system and maintained in an environment characterized by a strict 12‐h light cycle, a standard room temperature (21°C–26°C), and relative humidity (50–60%). Sterilized food and water ad libitum were provided. The mice were allowed to adapt to the environment before further experiments. All mice were euthanized by cervical dislocation.

### Natural products

Sesamin (CAS: 607‐80‐7; Cat# S890228), Resveratrol (CAS: 501‐36‐0; Cat# R817263), Proanthocyanidins (CAS: 4852‐22‐6; Cat# P823328), Baicalein (CAS: 491‐67‐8; Cat# B802463), Sanguinarine chloride (CAS: 5578‐73‐4; Cat# S817437), Quercetin (CAS: 117‐39‐5; Cat# Q817162), Plumbagin (CAS: 481‐42‐5; Cat# P832413) and Luteolin (CAS: 491‐70‐3; Cat# L812409) were purchased from Shanghai Macklin Biochemical Co., Ltd. Sodium houttuyfonate (CAS: 1847‐58‐1; Cat# S56908) and Umbelliferone (CAS: 93‐35‐6; Cat# S31317) were purchased from Shanghai Yuanye Bio‐Technology Co., Ltd. (Shanghai, China). Isochlorogenic acid A (CAS: 2450‐53‐5; Cat# A419565‐0100) was purchased from Sangon Biotech Co., Ltd. Ginkgetin (CAS: 481‐46‐9; Cat# BP0642) was purchased from Chengdu Biopurify Phytochemicals Ltd.

### Mouse natural products supplementation model

Seventy‐eight Balb/c female mice were randomly and equally divided into 13 cages with six mice per cage. The mice were acclimatized for 7 days under standard conditions. The control group (1 cage) received daily gavage of 200 μL of a solvent mixture, while the experimental groups (12 cages) were gavaged daily with 200 μL of a natural product dissolved in the solvent mixture. Each cage corresponded to a specific natural product. Gavage continued for 5 days. Following the final gavage, sterile fecal samples were collected from each cage for 16S rRNA gene sequence analysis. On Day 6, CRKP (OD_600_ = 2; 50 μL per mouse, 6.7–7.5 × 10^9^/mL CFU) was infected by intranasal delivery, and fresh lung tissue was harvested 6 h post‐infection. One portion of the lung tissue was fixed in 4% paraformaldehyde, while the other portion was weighed, mixed with 1 mL of PBS, and processed in a tissue grinder for 10 min. After grinding, 100 μL of the lung suspension was aspirated and dropped onto an LB culture plate by serial dilution, and incubated at 37°C for 12 h for colony counting. According to the results of colony counting, the experimental groups with significantly lower colony counts than the control group (C group) were labeled as effective groups (E group), and those with significantly higher colony counts than the control group were labeled as ineffective groups (N group). Related to Figure 1B.

The dose of Plumbagin was 10 mg/kg, the dose of Sanguinarine chloride was 40 mg/kg, and the dose of the rest of the natural products was 60 mg/kg. All natural products were solubilized with mixed solvents (10% DMSO + 40%PEG300 + 5%Tween‐80 + 45%Saline). The doses of the natural products used have been proven to be harmless to the health of mice by preliminary experiments.

### Mouse fecal microbiota transplantation (FMT) model

Twenty‐four Balb/c female mice were randomly selected to be kept normally for 7 days to acclimatize. Subsequently, all mice were fed drinking water containing a combination of antibiotics (1 g/L streptomycin, 0.5 g/L ampicillin, 1 g/L gentamicin, and 0.5 g/L vancomycin) for 5 days to clear the gut microbiota, and then normal drinking water was given for 2 days to allow for clearance of antibiotics from the intestinal tract. These mice were then randomly divided into three groups (*n* = 8): FMT‐C group (gavage of feces from donor group C), FMT‐N group (gavage of feces from donor group N), and FMT‐E group (gavage of feces from donor group E). Sesamin, isochlorogenic acid A, and sodium houttuyfonate were all included in the donor treatment regimen for FMT‐E. Umbelliferone, baicalein, and Sanguinarine chloride were all included in the donor treatment regimen for FMT‐N. FMT lasted from Day 7 to Day 12. CRKP (OD_600_ = 2; 50 μL per mouse) was infected by intranasal delivery on Day 13. Related to Figure 1F.

After the last FMT, sterile feces were collected for bacterial quantification. 6 h after CRKP infection, duodenal, colon, and some lung tissues were collected and stored at −80°C, 4% paraformaldehyde, and 2.5% glutaraldehyde for subsequent assays, respectively. The remaining lung tissues were collected for drop plate counting as described above.

### Mouse *Lactobacillus*/HK‐*L. m*/UL‐*L. m* supplementation model

Eighty Balb/c female mice were randomly divided into five groups (*n* = 16): control group (Con) and *Lactobacillus* supplement groups (*L. m* group/*L. r* group/*L. t* group/*L. j* group). Subsequently, adaptation was carried out for 7 days. 200 μL *Lactobacillus* (2.5 × 10^8^ CFU/mL) was gavaged to each mouse in the experimental groups, and 200 μL PBS was gavaged to each mouse in the Con group for 7 days. CRKP (OD_600_ = 2; 50 μL per mouse) infection was performed on Day 8. Six hours after infection, samples were collected, and lung colonization was counted as previously described. Related to Figure 3C.

*L. m*‐SNF group (*n* = 12), HK‐*L. m* group (*n* = 10), HK‐*L. m* ^Cells^ group (*n* = 12), HK‐*L. m* ^SNF^ group (*n* = 12), and UL‐*L. m* group (*n* = 12) were infected with CRKP (OD_600_ = 2, 50 μL per mouse) after gavage as previously described, and then the lung tissue was collected for drop plate counting. Related to Figure 4A–B.

HK‐*L. m*, HK‐*L. r*, HK‐*L. t* and HK‐*L. j* were administered to mice by intranasal delivery (OD_600_ = 2, 50 μL per mouse), gavage (OD_600_ = 2, 200 μL per mouse), and intraperitoneal injection (OD_600_ = 0.5, 100 μL per mouse), respectively. Lung tissues were then collected and drop‐plate counted after infection with CRKP (OD_600_ = 2, 50 μL per mouse) as described previously. Related to Figure S5A.

Sixty Balb/c female mice were randomly divided into five groups (*n* = 12): control group (Con) and experimental groups (HK‐*L. m* group, HK‐*L. m* ^DNA^ group, HK‐*L. m* ^RNA^ group, and HK‐*L. m* ^Protein^ group). Subsequently, adaptation was carried out for 7 days. 50 μL of enzymatically digested HK‐*L. m* was administered to each mouse in the experimental groups by intranasal delivery, and 50 μL PBS was administered to each mouse in the Con group by intranasal delivery for 7 days. Samples were collected after infection with CRKP (OD_600_ = 2, 50 μL per mouse), and lung tissue droplet plate counts were performed as described previously. Related to Figure 4D.

UL‐*L. m* ^DNA^, UL‐*L. m* ^RNA^, and UL‐*L. m* ^Protein^ were administered to mice by intranasal delivery (OD_600_ = 2, 50 μL per mouse) and gavage (OD_600_ = 2, 200 μL per mouse), respectively. Lung tissues were then collected and drop‐plate counted after infection with CRKP (OD_600_ = 2, 50 μL per mouse) as described previously. Related to Figure S5E,F.

HK‐*L. m*: *L. m* was inactivated by pasteurization for 15 min at 85°C. UL‐*L. m*: *L. m* was crushed by an ultrasonic crusher for 10 min (35%W, 5 s/5 s). For DNase I, RNase A, and proteinase K treatment of HK‐*L. m* and UL‐*L. m*, DNase I (Cat#RT411; TIANGEN) was incubated with HK‐*L. m* and UL‐*L. m* at 37°C for 20 min and then heat‐inactivated at 75°C for 10 min. RNase A (Cat# RT405; TIANGEN) was incubated with HK‐*L. m* and UL‐*L. m* at 37°C for 30 min and then heat‐inactivated at 85°C for 15 min. Proteinase K (Cat#RT403; TIANGEN) was incubated with HK‐*L. m* and UL‐*L. m* at 37°C for 20 min and then heat‐inactivated at 75°C for 10 min. Protein integrity was validated by 10% (w/v) SDS‐PAGE gel electrophoresis. DNA and RNA concentrations were measured with a NanoDrop 2000 UV–Vis spectrophotometer (Thermo Scientific).

### Mouse *L. m*‐MVs supplementation model

50 μL of *L. m*‐MVs (BCA: 1.03 mg/ml) and PBS were administered to each mouse in the *L. m*‐MVs group (*n* = 16) and Con group (*n* = 16) by intranasal delivery, respectively, for 7 consecutive days. Samples were collected after infection with CRKP (OD_600_ = 2, 50 μL per mouse), and lung tissue droplet plate counts were performed as described previously. Related to Figure 4M.

300 μL *L. m*‐MVs (BCA:1.4 mg/mL) and PBS were administered intragastrically to mice in the *L. m*‐MVs group (*n* = 16) and the Con group (*n* = 16), respectively, for 7 consecutive days. Samples were collected after infection with CRKP (OD_600_ = 2, 50 μL per mouse), and lung tissue droplet plate counts were performed as described previously. Related to Figure S5K.

### Mouse CRKP chronic infection model

Thirty Balb/c female mice were randomly selected to be kept normally for 7 days to acclimatize. All mice were infected with CRKP every 2 days for 2 weeks by intranasal delivery. From days 11 to 14, gavage of *L. m* and HK‐*L. m* or intranasal drops of MVs. Subsequently, following the final CRKP (OD_600_ = 2, 50 μL per mouse) infection, lung tissues were collected and processed for plate counting according to the method described previously. Related to Figure S7A.

### Bacterial strains and culture medium

The CRKP strain (designation: [C39]) used in this study was isolated from a sputum sample of a male infant patient admitted to the Intensive Care Unit of Central China Fuwai Hospital Affiliated to Zhengzhou University [59]. The patient was diagnosed with congenital heart disease and exhibited clinical symptoms of lower respiratory tract infection, and strain C39 was clinically associated with this infectious condition. *Lactobacillus reuteri* (*L. r*), *Lactobacillus taiwanensis* (*L. t*), *Lactobacillus johnsonii* (*L. j*), and *Lactobacillus murinus* (*L. m*) were isolated from the contents of the mouse cecum. Luria‐Bertani broth (LB, HB0128) medium and De Man, Rogosa and Sharpe broth (MRS, HB0384) medium were purchased from Qingdao Hope Bio‐technology.

### Isolation and characterization of *Lactobacillus*

The contents of the cecum of group E mice were collected in 1.5 mL EP tubes, 1 mL of sterile anaerobic PBS was added, and the homogenizer was used to grind for 5 min and then tenfold diluted. Then, 50 µL of the dilution was spread on MRS agar plates containing calcium carbonate. Plates were incubated anaerobically at 37°C for 24 h. The single colonies obtained were purified twice, and then Gram‐stained. Based on the results of Gram staining, DNA of Gram‐positive bacteria was extracted for 16S genus identification. A total of four species of *Lactobacillus reuteri* (*L. r*), *Lactobacillus taiwanensis* (*L. t*), *Lactobacillus johnsonii* (*L. j*), and *Lactobacillus murinus* (*L. m*) were identified.

### Cell lines

RAW264.7 cells were cultured in complete DMEM medium (Gibco) supplemented with 10% FBS and 1% penicillin/streptomycin at 37°C with 5% CO_2_.

### Histopathological analysis

Samples of the duodenum, colon, and lung were dewaxed with xylene, dehydrated in graded ethanol solutions, and embedded in paraffin using a fully enclosed tissue processor (ASP300S, Leica Biosystems). Intestinal sections (4 μm) and lung sections (3 μm) were mounted on glass slides, air‐dried at room temperature for 2 h, and stained with hematoxylin and eosin (H&E). H&E pictures were taken using an optical microscope (Leica 1100). An automatic best fit was applied for the best contrast.

Histopathological scoring of the duodenum was performed according to three indicators: villus height, crypt depth, and mucus thickness. Each of the three criteria was scored from 0 to 3 points, and the sum of the scores was calculated. The rules for scoring are described in detail in Table S1.

### TUNEL staining

Dewax the embedded paraffin sections, add 100 μL of proteinase K (20 µg/mL), and incubate at room temperature for 10 min. Wash with PBS, then treat with an appropriate amount of membrane lysis buffer (0.5% Triton X‐100) at room temperature for 20 min. Wash with PBS, incubate with Equilibration Buffer at room temperature for 10 min, and incubate with TdT incubation buffer (Cat# G1504; Servicebio) at 37°C in the dark for 1 h. Nuclei were stained with DAPI (2 µg/mL), coverslips were sealed with antifade mounting medium, and final images were captured under a microscope.

### Scanning electron microscope (SEM)

*L. m* and HK‐*L. m* bacterial precipitates were washed twice with 0.1 M phosphate buffer (pH 7.4) and then fixed overnight with 2.5% glutaraldehyde. After dehydration in a graded ethanol series (50%–100%), samples were critical‐point‐dried with CO_2_, mounted on stubs, and coated with 10 nm gold. Imaging was performed using a JEOL JSM‐7900F SEM at 5 kV with a working distance of 10 mm.

### Transmission electron microscope (TEM)

Fresh tissue samples and cell samples were fixed in 2.5% glutaraldehyde in 0.1 M phosphate buffer (pH 7.4) at 4°C overnight, then post‐fixed in 1% osmium tetroxide for 2 h. After dehydration through a graded ethanol series (30%–100%), the samples were infiltrated with EPON 812 resin and polymerized at 70°C for 24 h. The samples were sectioned using a Leica EM UC7 ultramicrotome to obtain ultrathin sections of 70 nm. These sections were then stained with uranyl acetate and lead citrate. After staining, the sections were air‐dried for observation under a low‐voltage transmission electron microscope (JEM‐1200EX).

### Cytokine expression analyses

Enzyme‐linked immunosorbent assay (ELISA) kits (MLbio, Shanghai, China) were used to quantify cytokines (TGF‐β, ml057830; IL‐22, ml063138; IL‐1β, mL098416; IL‐6, mL098430; TNF‐α, mL002095; IFN‐γ, mL002277; IL‐4, mL064310; IL‐10, mL037873) levels. 100 mg of lung tissue samples were minced and then mixed with 1 mL of pre‐chilled PBS buffer. The mixture was homogenized at 4°C and 60 Hz for 10 min. The homogenate was centrifuged at 5000 × *g* for 15 min, and the supernatant was collected for detection according to the manufacturer's instructions.

### Fecal bacteria quantification

*L. m* cells in the logarithmic growth phase were processed for DNA extraction using the Microbiome DNA Isolation Kit (Cat# DC502‐01; Vazyme). DNA was diluted 10‐fold serially five times. qPCR was conducted using the diluted DNA with *L. m*‐16S rRNA primers (Table S2) and SYBR qPCR Master Mix (Cat# Q312‐02; Vazyme). DNA concentration was converted to copy numbers, and a linear regression analysis was performed to correlate copy numbers with CT values.

Fecal bacterial DNA was isolated using a TIANamp Stool DNA Kit (Cat# DP328‐02; TIANGEN). Fecal bacteria were quantified by qPCR using the *L. m*‐ specific 16S rRNA primers. The resulting CT values were used to determine the corresponding bacterial copy numbers from the linear regression curves established previously.

### Extraction and characterization of *L. murinus* membrane vesicles

*Lactobacillus murinus* was cultured in MRS broth at 37°C under anaerobic conditions until reaching the mid‐log phase. Bacterial cells were removed by centrifugation at 10,000 *g* for 30 min at 4°C, followed by filtration of the supernatant through a 0.22 μm membrane to eliminate residual cells. The clarified supernatant was concentrated using ultrafiltration (100 kDa cutoff Amicon filters). MVs were pelleted by ultracentrifugation at 200,000 *g* for 2 h at 4°C, washed with PBS, and resuspended in the same buffer. Protein concentration was quantified by BCA assay, and purity was confirmed by transmission electron microscopy (TEM) and nanoparticle tracking analysis (NTA).

### RT‐qPCR

Total miRNA was extracted from the MVs, cells, and lungs using the Magnetic miRNA Kit (Cat# ES301; TIANGEN). Total mRNA was extracted from the cells and lungs using RNA Easy Fast Kit (Cat# DP451; TIANGEN). The RNA quality was quantified by the ratio of absorbance (260 nm/280 nm) using a NanoDrop spectrophotometer (Thermo Scientific). The first‐strand cDNA from miRNA was synthesized using the Mir‐X^TM^ miRNA First‐Strand Synthesis Kit (Cat# 638313; Takara). The first‐strand cDNA from mRNA was synthesized using the HiScript III RT Supermix for qPCR with gDNA wiper (Vazyme). After synthesizing cDNAs, SYBR qPCR Master Mix was used to perform real‐time PCR. RT‐qPCR test was performed using a 7500 Fast Real‐Time PCR System (Applied Biosystems). The fold changes of gene expression were determined using the 2^−ΔΔCt^ method. *β‐actin* mRNA levels and *U6* miRNA levels were used as the internal controls. The gene sequences were designed by PrimerBank and synthesized by Youkang Biological Technology Co. Ltd. The primers utilized in this study are listed in Tables S2, 3.

### Digital PCR

Digital PCR (dPCR) was performed on the QIAcuity dPCR System with the QIAcuity Probe PCR Kit. The 40 μL reaction system consisted of 10 μL of 4 × QIAcuity Probe PCR Master Mix, 4 μL of 10 × primer‐probe mix (final concentrations: 800 nM for forward and reverse primers, 400 nM for probe), 15 μL of cDNA template, and 11 μL of nuclease‐free water. Reaction mixtures were prepared in 96‐well plates, transferred to QIAcuity Nanoplate 26 K, and sealed with specialized film. The instrument automatically completed sample partitioning, thermal cycling, and imaging, generating approximately 26,000 nanopartitions per well. The thermal cycling protocol was as follows: initial denaturation at 95°C for 2 min, 40 cycles of 95°C for 15 s and 60°C for 30 s, and a final cooling step at 40°C for 5 min. Fluorescence signals were collected via the FAM channel (excitation: 463–503 nm, emission: 518–548 nm), with other channels closed. Raw data were analyzed using QIAcuity Software Suite, and the absolute abundance of mim‐miR‐21‐5p was quantified as copies/μL.

### Conventional PCR amplification

After reverse transcribing MVs‐derived miRNAs into cDNA using the poly(A) polymerase, the specific cDNA fragments of the miRNA were amplified using 2 × Magic Green Taq SuperMix (Cat# 21502; TOLOBIO) and specific primers (Table S3). After PCR amplification, 0.4% agarose gel electrophoresis was used for target miRNA qualitative detection. 10 μL of PCR product was loaded into gel wells. Run electrophoresis at 100 V for 30 min in 1 × TAE buffer. Visualize the gel under UV light using Tanon‐4600.

### Western blot analysis

Total proteins from cells were extracted using Cell Complete Lysis Buffer for Western and IP (Cat# P0037, Beyotime) following the manufacturer's protocols. The total protein content was determined using a BCA protein assay kit (Cat# P0012, Beyotime). Equal amounts of protein (40 μg) were electrophoresed on 4–20% (w/v) SDS–PAGE gels and then transferred to PVDF membranes. Next, the membranes were probed overnight at 4°C with primary antibodies against PDCD4 (Cat# A01105‐4; BOSTER), Nrf2 (Cat# WL02135; Wanleibio), SOD2 (Cat# BA4566 BOSTER), Arg‐1(Cat# A01106 BOSTER), iNOS (Cat# BA0362 BOSTER) and β‐actin (Cat# ab8227; Abcam). Then, the membranes were washed with TBST for 30 min, and the secondary antibody (Cat# RGAR001; Proteintech) was applied and incubated for 1 h at room temperature. The membranes were again washed with TBST for 30 min, and images were captured with an imaging system (AI600, CE). All blots were derived from the same experiment and processed in parallel. Finally, the gray level of the exposed strip was analyzed using ImageJ software (v1.8.0, NIH).

### Live imaging

Isolated MVs (BCA: 1 mg/mL) were incubated with the lipophilic near‐infrared dye DIR (MCE, Cat# HY‐D1028; 10 μM) at 37°C for 30 min protected from light. Unbound dye was removed using membrane vesicle‐free dye removal centrifugal columns (Cat# E30038; Mele Biotech). The labeled MVs (DIR‐MVs) were resuspended in sterile PBS. Balb/c female mice were administered intragastrically with 400 μg of DIR‐MVs in 300 μL PBS, or an equal volume of DIR dye as the negative control. In vivo imaging was performed using a PerkinElmer (IVIS Lumina III) at different time points, and representative images were taken.

### Luciferase assay

Cells were uniformly seeded in 96‐well plates. Prepare the transfection mixture using Opti‐MEM (Cat# 31985‐062; Gibco), lipo3.0 (Cat# BMU111‐CN; Abbkine), plasmid (PDCD4‐MUT and PDCD4‐WT; KeyGEN BioTECH), and RNA (mim‐miR‐21a‐5p mimics and NC mimics; Gene Adv), then incubate at room temperature for 30 min. Aspirate the original medium from the 96‐well plate, add the transfection complex, and incubate at 37°C for 24 h. Process cells using the KeyGEN Firefly‐Renilla Luciferase Detection Kit (Cat# KGE3308; KeyGEN BioTECH) according to the manufacturer's instructions, and detect the firefly and Renilla luminescence intensity. The sequences utilized in this study are listed in Table S4.

### Immunofluorescence

MVs were labeled with the PKH67 fluorescent probe using the BeyoExo™ Exosome Labeling and Tracing Kit (Cat# C3635M; Beyotime) and then incubated with RAW264.7 cells overnight. On prepared slides, cells were fixed with 4% paraformaldehyde for 10 min at room temperature, washed for 5 min, and then permeabilized with 0.5% Triton X‐100 for 5 min. After 5 min of washing, cell membranes were stained with TRITC Phalloidin (Cat# CA1610; Solarbio) for 30 min protected from light, and nuclei were stained with Hoechst 33342 (Cat# C1022; Beyotime) for 10 min protected from light. After dropping the anti‐fluorescence quenching sealer (Cat# G1401; Servicebio), a CLSM microscope (Leica TCS SP2) was used to detect and capture images. The resulting fluorescence was characterized by a blue nucleus in the Hoechst 33342 channel, green signal in the PKH67 channel, and red in the TRITC Phalloidin channel.

FAM‐labeled mim‐miR‐21a‐5p (20 μM; Cat# GAF712299; Gene Adv) was gently mixed with 150 μL of MVs to a final concentration of 3 μM, followed by ultrasonic treatment on ice for 4 min (35 W, 2 s sonication, 8 s interval). Then, the mixture was incubated at 37°C for 30 min in the dark. Unloaded free small RNAs were removed using a free small RNA removal column (Cat# E0051; Mele Biotech). Subsequently, MVs loaded with FAM‐mim‐miR‐21a‐5p were labeled with DIR, and unbound free DIR dye was removed using a free dye removal column (Cat# E30038; Mele Biotech). After dropping anti‐fluorescence quenching mounting medium onto the prepared slides, detection and image capture were performed using a CLSM microscope. The resulting fluorescence was characterized by a red signal in the DIR channel and a green signal in the FAM‐mim‐miR‐21a‐5p channel.

After MVs loaded with FAM‐mim‐miR‐21a‐5p were co‐incubated with RAW264.7 cells for 8 h, detection and image capture were performed using a CLSM microscope according to the aforementioned method.

### mim‐miR‐21a‐5p inhibition and overexpression

mim‐miR‐21a‐5p inhibitor and NC inhibitor (Cat# GAF712286 and GAmir700002, Gene Adv) according to the aforementioned method were separately loaded into MVs, and then co‐incubated with RAW264.7 cells overnight. After incubation, the cells were infected with CRKP (MOI = 10) for 1 h; extracellular CRKP was eliminated with gentamicin (200 μg/mL), and the cells were collected for plating and counting to determine the intracellular bacterial load.

RAW264.7 cells were seeded in 24‐well plates at a density of 1 × 10^5^ cells per well 1 day prior to transfection to achieve 60–80% confluence. Prior to transfection, 50 μL Opti‐MEM reduced serum medium was used to dilute 10 pmol miRNA (mim‐miR‐21a‐5p mimics and NC mimics; Cat# GAF711093 and GAmir700001, Gene Adv) and 3 μL Lipofectamine® RNAiMAX Reagent (Cat# 13778030, Invitrogen). Diluted miRNA and Lipofectamine® RNAiMAX Reagent were incubated for 5 min at room temperature to allow complex formation. The culture medium from the cells was replaced with the miRNA‐Lipofectamine® RNAiMAX complexes dropwise. At 36 h post‐transfection, the cells were infected with CRKP (MOI = 10) for 1 h, and the intracellular bacterial load was determined by plating and counting. Meanwhile, cellular RNA and protein were extracted to verify the transfection efficiency. The sequences utilized in this study are listed in Table S4.

### Oxidative phosphorylation inhibition assay

RAW264.7 cells were seeded into 24‐well plates. When the cell confluency reached 60%–80%, rotenone (Cat# R426175, Aladdin) was added to the experimental group to a final concentration of 50 nM, while an equal volume of DMSO solvent was added to the control group, and all groups were co‐incubated with the cells for 2 h. Subsequently, MVs were added for co‐incubation for 8 h, followed by infection with CRKP (MOI = 10) for 1 h. The cells were collected for plating and counting to determine the intracellular bacterial load, and cellular RNA and protein were extracted for downstream experimental evaluation.

### PDCD4 knockout strain of RAW264.7 cells

PDCD4 knockout strain of RAW264.7 cells (Cat# YKO‐M270) was purchased from UBIGENE Biology. The PDCD4 knockout cells (*pdcd4* ^−/−^) and wild‐type cells (WT) were separately seeded into 24‐well plates. When the cell confluency reached 60%–80%, MVs were added for co‐incubation for 8 h, followed by infection with CRKP (MOI = 10) for 1 h. The cells were collected, and the intracellular bacterial load was determined by plating and counting. Meanwhile, cellular RNA and protein were extracted for downstream experimental evaluation.

### 16S rRNA sequencing

Three fecal samples were collected from mice in each group after the last gavage of the natural product of the natural product supplementation model. The samples were then processed to isolate total microbial genomic DNA using the DNeasy PowerSoil Pro Kit (Qiagen) according to the manufacturer's instructions. DNA concentration and purity were measured with a NanoDrop 2000 UV–Vis spectrophotometer (Thermo Scientific), and integrity was verified by 1% agarose gel electrophoresis. The V3 and V4 hypervariable regions of the bacterial 16S rRNA gene were amplified using primers 338 (forward: 5'‐ACTCCTACGGGGAGGCAGCAG‐3') and 806 (reverse: 5'‐GGACTACHVGGGGTWTCTAAT‐3'). Amplicons were resolved on a 2% agarose gel, gel‐purified with the AxyPrep DNA Gel Extraction Kit (Axygen Biosciences), and quantified using QuantiFluor™‐ST (Promega). Purified products were pooled in equimolar amounts and paired‐end sequenced (2 × 300) on an Illumina MiSeq platform (Illumina) according to the standard protocols by Majorbio Bio‐Pharm Technology Co. Ltd. All raw sequencing data have been deposited in the NCBI Sequence Read Archive under accession number PRJNA1346086.

Raw FASTQ files were demultiplexed, quality‐filtered by Trimmomatic, and merged by FLASH with the following criteria: (i) The reads were truncated at any site receiving an average quality score <20 over a 50 bp sliding window. (ii) Primers were exactly matched, allowing 2 nucleotide mismatches, and reads containing ambiguous bases were removed. (iii) Sequences whose overlap was longer than 10 bp were merged according to their overlap sequence. Operational taxonomic units were clustered with a 97% similarity cutoff using UPARSE (version 7.1 http://drive5.com/uparse/), and chimeric sequences were identified and removed using UCHIME. The taxonomy of each 16S rRNA gene sequence was analyzed by the RDP Classifier algorithm (http://rdp.cme.msu.edu/) against the Silva (SSU123) 16S rRNA database using a confidence threshold of 70%.

The 16S sequencing‐related data were analyzed as follows: Gene Cloud tools (https://www.genescloud.cn) were used to calculate the Alpha diversity index. Gene Cloud tools (https://cloud.majorbio.com) were used to calculate the PCoA analysis (Principal Coordinate Analysis), phylum‐level analysis, genus‐level analysis, LEfSe analysis (Linear discriminant analysis Effect Size), and ternary diagram analysis. Gene Cloud tools (https://www.omicshare.com/) were used to calculate the random forest analysis and ROC analysis.

### Small RNA sequencing

Total RNA was extracted from all samples using the miRNeasy kit (Qiagen) according to the manufacturer's instructions. Then RNA quality was determined by a 5300 Bioanalyser (Agilent) and quantified using the ND‐2000 (NanoDrop Technologies). RNA purification, reverse transcription, library construction, and sequencing were performed by Shanghai Majorbio Bio‐pharm Biotechnology Co., Ltd., following standard protocols. A total amount of 1 μg of total RNA per sample was used as input material for the small RNA library. Sequencing libraries were generated using QIAseq miRNA Library Kit (Qiagen) following the manufacturer's recommendations. The activated 5' and 3' adaptors were ligated to the total RNA, respectively. Then the adaptor‐ligated RNA was transcribed into first‐strand cDNA using reverse transcriptase and a random primer. A PCR reaction was performed using primers complementary to the adaptors for 11–12 cycles; fragments of appropriate size were isolated by a 6% Novex TBE PAGE gel. After quantification using the Qubit 4.0 fluorometer, the sequencing library was loaded onto the NovaSeq X Plus platform for sequencing using the NovaSeq Reagent Kit. Raw reads in FASTQ format were processed through Fastx‐Toolkit with default parameters. After this step, clean reads were obtained by removing the 3' end adapter, reads containing poly‐N, low‐quality bases (Sanger base quality of <20) at the 3' end, and sequencing adapters from raw data with the FastX Toolkit software. All identical sequences of sizes ranging from 18 to 32 nt were counted and eliminated from the initial dataset.

Their sequence similarity to mouse microRNAs (mus‐miRNAs) was analyzed using the miRBase database (http://www.mirbase.org/). The BLASTN tool was used to align the bacterial sRNA dataset with reference sequences of mus‐miRs. The expression level of each candidate bacterial sRNA was calculated according to the transcripts per million reads (TPM) method. Differential expression analysis was performed using DESeq. 2. Target gene predictions of miRNAs were performed with miRWalk and TargetScan. The predicted target genes were annotated with the GO (http://www.geneontology.org/) database. GO functional enrichment analysis was carried out by the Goatools software. The bacteria‐derived miRNAs mentioned in this study all refer to prokaryotic sRNAs that functionally mimic the mode of action of eukaryotic miRNAs.

### Single‐cell transcriptome sequencing

Single‐cell transcriptome sequencing was performed by a commercial company (GENE Denovo Biotech Co., Ltd.). Cellular suspensions were loaded on a 10× Genomics GemCode Single‐cell instrument that generates single‐cell Gel Bead‐in‐Emulsion (GEMs). Libraries were generated and sequenced from the cDNAs with Chromium Next GEM Single Cell 3' Reagent Kits v3.1. Upon dissolution of the Gel Bead in a GEM, primers containing (i) an Illumina® R1 sequence (read 1 sequencing primer), (ii) a 16 nt 10 × Barcode, (iii) a 10 nt Unique Molecular Identifier (UMI), and (iv) a poly‐dT primer sequence were released and mixed with cell lysate and Master Mix. Barcoded, full‐length cDNAs were then reverse‐transcribed from polyadenylated mRNA.

Silane magnetic beads were used to remove leftover biochemical reagents and primers from the post‐GEM reaction mixture. Full‐length, barcoded cDNAs were then amplified by PCR to generate sufficient mass for library construction. R1 (read 1 primer sequence) was added to the molecules during GEM incubation. P5, P7, a sample index, and R2 (read 2 primer sequence) were added during library construction via End Repair, A‐tailing, Adaptor Ligation, and PCR. The final libraries contained the P5 and P7 primers used in Illumina bridge amplification. High‐throughput sequencing was performed on the final libraries using Illumina NovaSeq X Plus.

The count quantification, genome alignment, and data quality control were performed through 10 × Genomics Cell Ranger software (version 8.0.0). In summary, a matrix of raw UMI counts was created, and reads with poor‐quality barcodes and UMIs were excluded. For UMI counting, reads that were uniquely aligned to the transcriptome and overlapped an exon by at least 50% were taken into account. Following quality assessment, gene expression was standardized using LogNormalize, a method for global scaling normalization. Afterward, we utilized a technique called Harmony to combine all the samples, thereby reducing any possible batch effects. Subsequently, principal component analysis was performed on the combined expression matrix to reduce dimensions.

### Cell clustering and annotation

Cells were clustered using Seurat. In brief, the cells were embedded in a shared nearest‐neighbor graph and partitioned into highly interconnected quasi‐populations based on the calculated distances between cells in the principal components. Subsequently, the Louvain method was employed to cluster and group the cells, and the results were visualized using t‐SNE plots. Cell types were annotated based on the expression of conserved lineage‐specific markers. After that, as previously documented, the annotation was manually adjusted using the conventional markers [60].

### GO enrichment analysis

All peak‐related genes were mapped to GO terms in the Gene Ontology database (http://www.geneontology.org/); gene numbers were calculated for every term, and significantly enriched GO terms in differentially expressed genes compared to the genome background were defined by a hypergeometric test. The calculating formula of *p*‐value is:

P=1−∑i=0m−1MiN−Mn−iNn.

Here, *N* is the number of all genes with GO annotation; *n* is the number of differentially expressed genes in *N*; *M* is the number of all genes that are annotated to a certain GO term; m is the number of differentially expressed genes in *M*. The calculated *p*‐values were subjected to FDR Correction, taking FDR ≤ 0.05 as a threshold. GO terms meeting this condition were defined as significantly enriched GO terms in differentially expressed genes. This analysis was able to recognize the main biological functions that differentially expressed genes perform.

### Pathway enrichment analysis

Pathway enrichment analysis identified significantly enriched metabolic pathways or signal transduction pathways in differentially expressed genes compared with the whole genome background. The calculating formula of *p*‐value is:

P=1−∑i=0m−1MiN−Mn−iNn.

Here, *N* is the number of all transcripts with KEGG annotation, *n* is the number of differentially expressed genes in *N*, *M* is the number of all transcripts annotated to specific pathways, and m is the number of differentially expressed genes in *M*. The calculated *p*‐value was subjected to FDR Correction, taking FDR ≤ 0.05 as a threshold. Pathways meeting this condition were defined as significantly enriched pathways in differentially expressed genes.

### Biochemical analysis

The concentration of α‐KG (Cat# BC5425) and the activity of SDH (Cat# BC0955), Complex III (Cat# BC3245), and Complex IV (Cat# BC0945) in cells were detected according to the manufacturer's instructions (Solarbio Biotechnology Co. Ltd.). The levels of JC‐1 (Cat# C2003S) and ROS (Cat# S0033S) in cells were detected according to the manufacturer's instructions (Beyotime Biotechnology Co. Ltd.). The levels of ATP and ADP in cells were detected according to the manufacturer's instructions (Cat# E‐BC‐F004; Elabscience Biotechnology Co. Ltd.).

### Flow cytometry assay

Cells were uniformly seeded in six‐well plates and incubated overnight with MVs (1 mg/mL), followed by infection with CRKP (MOI = 10) for 1 h. A portion of the infected cells was analyzed using a ROS assay kit (Cat# S0033S; Beyotime) according to the manufacturer's instructions to detect ROS levels. Another portion was incubated with Fc antibody (Cat# 101301), F4/80 antibody (Cat# 123119), CD11b antibody (Cat# 101211), CD86 antibody (Cat# 105013), and CD206 antibody (Cat# 141705), respectively, as per the protocols (Biolegend), to assess M2 polarization levels. 10,000 ungated events were measured using a CytExpert Flow Cytometer (Beckman, USA), and the results were analyzed using FlowJo software v10.8.1.

### *In vitro* antibacterial activity

The OD_600_ of CRKP and *L. m* in the logarithmic growth phase was adjusted to 0.5 using sterile PBS. CRKP and *L. m* were co‐cultured at various volume ratios in aerobic LB medium and anaerobic MRS medium, respectively. Plate counts were performed at different time points.

Growth curves were obtained based on the automated growth analysis system in a Microplate reader (Tecan). Add *L. m*‐MVs to LB medium at low (6.87 μg/mL), medium (13.7 μg/mL), and high (20.6 μg/mL) doses. CRKP suspension was diluted 1:1000 in pre‐prepared LB medium and added in triplicate to a 96‐well plate. The total volume was up to 200 μL. OD_600_ values were recorded automatically within 10 h.

MIC values were determined by the standard broth microdilution method. In brief, HK‐*L. m* (2560 μg/mL) and *L. m*‐MVs (2560 μg/mL) were twofold diluted in LB medium with a total volume of 100 μL and mixed with an equal volume of CRKP suspensions (approximately 1.0 × 10^5^–10^6^ CFU per mL) in a clear UV‐sterilized 96‐well microliter plate. After 18 h incubation at 37°C, the MIC values were defined as the lowest concentration of HK‐*L. m* or *L. m*‐MVs without any visible growth of bacteria.

### Human subjects

Fecal samples from healthy individuals and CRKP patients were used to analyze the relative abundance of *Lactobacillus* and the absolute expression level of *Lactobacillus murinus*. Detailed information on human samples is listed in Table S5.

### Statistical analysis

Statistical analyses were conducted using GraphPad Prism version 10.1.2 software. Unless otherwise specified, two‐tailed Student's *t* test (follows a normal distribution) and Mann–Whitney *U* test (does not follow a normal distribution) were used to compare two groups. One‐way ANOVA with Tukey's post hoc test (follows a normal distribution) and Kruskal–Wallis test with Dunn's post hoc test (does not follow a normal distribution) were used to compare three or more groups. Results are presented as mean ± SEM in plots with error bars, unless stated otherwise. The Majorbio Cloud Platform (https://cloud.majorbio.com/) and the Omic Share platform (https://www.omicshare.com/) were utilized for the analysis of omics sequencing data and the generation of heatmaps. Differences with *p* < 0.05 were considered significant. Significance levels were indicated by numbers of asterisks: *p < 0.05, **p < 0.01, ***p < 0.001, and ****p < 0.0001.

## AUTHOR CONTRIBUTIONS

**Tingting Xu**: Methodology; Validation; investigation; writing—original draft. **Xiuying Tao**: investigation; methodology; validation. **Dan Fang**: Methodology; validation. **Haijie Zhang**: Methodology; validation. **Zhiqiang Wang**: Funding acquisition; investigation. **Yuan Liu**: Conceptualization; investigation; supervision; funding acquisition; project administration; writing—review and editing.

## CONFLICT OF INTEREST STATEMENT

The authors declare no conflicts of interest.

## ETHICS STATEMENT

All experiments complied with institutional ethical guidelines and were specifically approved by the Jiangsu Administrative Committee of Laboratory Animals (No. SYXK‐2022‐0044) and the Yangzhou University Affiliated Hospital (No. 2025‐YKL09‐K16).

## Supporting information


**Figure S1:** Gut microbiota driven by natural products enhances lung resistance against CRKP infection, related to Figure 1.
**Figure S2:** Changes in the structure of the gut microbiota in the natural product supplementation model, related to Figure 2.
**Figure S3:** Analysis of gut microbiota in the natural product supplementation model, related to Figure 2J.
**Figure S4:** Stable colonization of *Lactobacillus murinus* enhances intestinal barrier function, related to Figure 3.
**Figure S5:** *Lactobacillus* protects against CRKP infection, related to Figure 4.
**Figure S6:** sRNA analysis of bacteria and lungs, related to Figure 5.
**Figure S7:** *L. m*, HK‐*L. m*, and *L. m*‐MVs exhibit no direct inhibitory effect on CRKP in vitro, related to Figure 5.
**Figure S8:** *PDCD4* suppression sustains OXPHOS via activating Nrf2/SOD2 pathway, related to Figure 6.
**Figure S9:** OXPHOS‐sustained M2 macrophage polarization enhances host resistance to CRKP infection, related to Figure 6.

**Table S1:** Histological damage score.
**Table S2:** Primer sequences of mRNA.
**Table S3:** Primer sequences of sRNA.
**Table S4:** Sequences of sRNA and plasmids.
**Table S5:** Clinical information of human subjects.

## Data Availability

16S rRNA gene sequencing data, small RNA sequencing data, single‐cell transcriptome data, and bacterial metagenomic sequencing have been deposited in the NCBI database (PRJNA1346086) https://www.ncbi.nlm.nih.gov/bioproject/PRJNA1346086. The data used are saved in GitHub (https://github.com/ting0916-bit/Liu-Yuan-imeta2026). Supplementary materials (figures, tables, graphical abstract, slides, videos, Chinese translated version, and updated materials) may be found in the online DOI or iMeta Science (http://www.imeta.science/).

## References

[1] Zhang, Feilong , Xinmeng Liu , Ziyao Li , Zhihua Li , Zichen Lei , Yanyan Fan , Xinrui Yang , et al. 2025. “Tracking International and Regional Dissemination of the KPC/NDM Co‐Producing *Klebsiella pneumoniae* .” Nature Communications 16: 5574. 10.1038/s41467-025-60765-7 PMC12216068 40593598

[2] Wang, Qi , Ruobing Wang , Shuyi Wang , Anru Zhang , Qiaoyan Duan , Shijun Sun , Longyang Jin , et al. 2024. “Expansion and Transmission Dynamics of High Risk Carbapenem‐Resistant *Klebsiella pneumoniae* Subclones in China: An Epidemiological, Spatial, Genomic Analysis.” Drug Resistance Updates 74: 101083. 10.1016/j.drup.2024.101083 38593500

[3] Holmes, Caitlyn L. , Katherine G Dailey , Karthik Hullahalli , Alexis E Wilcox , Sophia Mason , Bridget S Moricz , Lavinia V Unverdorben , et al. 2025. “Patterns of *Klebsiella pneumoniae* Bacteremic Dissemination From the Lung.” Nature Communications 16: 785. 10.1038/s41467-025-56095-3 PMC11742683 39824859

[4] Wang, Minggui , Michelle Earley , Liang Chen , Blake M Hanson , Yunsong Yu , Zhengyin Liu , Soraya Salcedo , et al. 2022. “Clinical Outcomes and Bacterial Characteristics of Carbapenem‐Resistant *Klebsiella pneumoniae* Complex Among Patients From Different Global Regions (CRACKLE‐2): A Prospective, Multicentre, Cohort Study.” The Lancet Infectious Diseases 22: 401–412. 10.1016/s1473-3099(21)00399-6 PMC8882129 34767753

[5] Jin, Xuting , Jiajia Ren , Ruohan Li , Ya Gao , Haoying Zhang , Jiamei Li , Jingjing Zhang , Xiaochuang Wang , and Gang Wang . 2021. “Global Burden of Upper Respiratory Infections in 204 Countries and Territories, From 1990 to 2019.” eClinicalMedicine 37: 100986. 10.1016/j.eclinm.2021.100986 PMC8343248 34386754

[6] Chen, Chih‐Yu , Kuang‐Yao Yang , Chung‐Kan Peng , Chau‐Chyun Sheu , Ming‐Cheng Chan , Jia‐Yih Feng , Sheng‐Huei Wang , et al. 2022. “Clinical Outcome of Nosocomial Pneumonia Caused By Carbapenem‐Resistant Gram‐Negative Bacteria in Critically Ill Patients: A Multicenter Retrospective Observational Study.” Scientific Reports 12: 7501. 10.1038/s41598-022-11061-7 PMC9079069 35525867

[7] Hu, Fupin , Yuqing Pan , Heng Li , Renru Han , Xiao Liu , Ruijing Ma , Yongqin Wu , et al. 2024. “Carbapenem‐Resistant *Klebsiella pneumoniae* Capsular Types, Antibiotic Resistance and Virulence Factors in China: A Longitudinal, Multi‐Centre Study.” Nature Microbiology 9: 814–829. 10.1038/s41564-024-01612-1 PMC10914598 38424289

[8] Chen, Jing , Binbin Xia , Yang Liu , Wenfang Sun , Fang Liu , Jingyao Pang , and Hua Cheng . 2024. “Clinical Outcomes and Safety of Polymyxin B Versus Tigecycline Combination Therapy for Pneumonia of Carbapenem‐Resistant *Klebsiella pneumoniae*: A Retrospective Cohort Study.” Annals of Medicine 56: 2397087. 10.1080/07853890.2024.2397087 PMC11382689 39239861

[9] Wu, Yuchen , Fan Pu , Zelin Yan , Yanyan Zhang , Kaichao Chen , Shengkai Li , Yuezhuo Wang , et al. 2025. “Geographic Containment and Virulence‐Resistance Trade‐Offs Drive the Evolution of Hypervirulent *Klebsiella pneumoniae* .” iMeta 4: e70077. 10.1002/imt2.70077 PMC12528002 41112046

[10] Fröding, Inga , Sophia David , Corin Yeats , Khalil Abu‐Dahab , Barbara Albiger , Nabil‐Fareed Alikhan , Erik Alm , et al. 2026. “Persistent Spread of Carbapenemase‐Producing *Klebsiella pneumoniae* in Acute Care Hospitals in 36 European Countries (The CCRE Survey): A Prospective, Multicentre, Cross‐Sectional, Epidemiological, Microbiological, and Genomic Surveillance Study.” The Lancet Microbe 7: 101320. 10.1016/j.lanmic.2025.101320 PMC13259977 42127954

[11] Li, Xi , Xinhong Han , Xinyan Tang , Zhaokun Fan , Li Ding , Ronghua Geng , Qiong Chen , et al. 2026. “Clinical Characteristics and Molecular Epidemiology of KPC‐NDM Co‐Producing Carbapenem‐Resistant *Klebsiella pneumoniae* in China: A Multicentre Retrospective Case‐Control Study.” eBioMedicine 126: 106222. 10.1016/j.ebiom.2026.106222 PMC13015248 41861520

[12] Sati, Hatim , Elena Carrara , Alessia Savoldi , Paul Hansen , Jacopo Garlasco , Enrica Campagnaro , Simone Boccia , et al. 2025. “The WHO Bacterial Priority Pathogens List 2024: A Prioritisation Study to Guide Research, Development, and Public Health Strategies Against Antimicrobial Resistance.” The Lancet Infectious Diseases 25: 1033–1043. 10.1016/s1473-3099(25)00118-5 PMC12367593 40245910

[13] Bublitz, Arne , Madita Brauer , Stefanie Wagner , Walter Hofer , Mathias Müsken , Felix Deschner , Till R Lesker , et al. 2023. “The Natural Product Chlorotonil A Preserves Colonization Resistance and Prevents Relapsing Clostridioides Difficile Infection.” Cell Host & Microbe 31: 734–750.e8. 10.1016/j.chom.2023.04.003 37098342

[14] Deschner, Felix , Dietrich Mostert , Jan‐Martin Daniel , Alexander Voltz , Dana Carina Schneider , Navid Khangholi , Jürgen Bartel , et al. 2025. “Natural Products Chlorotonils Exert a Complex Antibacterial Mechanism and Address Multiple Targets.” Cell Chemical Biology 32: 586–602.e15. 10.1016/j.chembiol.2025.03.005 40203831

[15] Zhao, Wen‐Bin , Jun‐Xia An , Ya‐Rui Jin , Chen‐Xin Jing , Shao‐Yong Zhang , Hong‐Jie Liang , Tian‐Li Dai , et al. 2025. “Indoloquinoline Alkaloid Neocryptolepine Derivative Inhibits Botrytis Cinerea by Targeting Thiamine Thiazole Synthase.” Science Advances 11: eadq5329. 10.1126/sciadv.adq5329 PMC11900860 40073123

[16] Zhao, Yingying , Jinfeng Wei , Changqin Li , Adel F Ahmed , Zhenhua Liu , and Changyang Ma . 2022. “A Comprehensive Review on Mechanism of Natural Products Against *Staphylococcus aureus* .” Journal of Future Foods 2: 25–33. 10.1016/j.jfutfo.2022.03.014

[17] Li, Jianguo , Tingyin Lu , Yuefei Chu , Yuejun Zhang , Jing Zhang , Wenzhen Fu , Jian Sun , et al. 2024. “Cinnamaldehyde Targets SarA to Enhance β‐lactam Antibiotic Activity Against Methicillin‐Resistant *Staphylococcus aureus* .” mLife 3: 291–306. 10.1002/mlf2.12121 PMC11211666 38948140

[18] Dai, Huanqin , Junjie Han , Tao Wang , Wen‐Bing Yin , Yihua Chen , and Hongwei Liu . 2023. “Recent Advances in Gut Microbiota‐Associated Natural Products: Structures, Bioactivities, and Mechanisms.” Natural Product Reports 40: 1078–1093. 10.1039/d2np00075j 37013809

[19] Mastrangelo, Annalaura , Iñaki Robles‐Vera , Diego Mañanes , Miguel Galán , Marcos Femenía‐Muiña , Ana Redondo‐Urzainqui , Rafael Barrero‐Rodríguez , et al. 2025. “Imidazole Propionate Is a Driver and Therapeutic Target in Atherosclerosis.” Nature 645: 254–261. 10.1038/s41586-025-09263-w PMC12408353 40670786

[20] Liu, Jiaqi , Youhua Liu , Chaoqun Huang , Chuan He , Tongyudan Yang , Ruiti Ren , Zimeng Xin , and Xinxia Wang . 2025. “Quercetin‐Driven *Akkermansia muciniphila* Alleviates Obesity by Modulating Bile Acid Metabolism Via an ILA/m(6)A/CYP8B1 Signaling.” Advanced Science 12: e2412865. 10.1002/advs.202412865 PMC11948036 39888270

[21] Wu, Hao , Yao‐Lei Li , Yu Wang , Yu‐Ge Wang , Jia‐Hui Hong , Mi‐Mi Pang , Pan‐Miao Liu , and Jian‐Jun Yang . 2025. “Anemoside B4 Alleviates Ulcerative Colitis by Attenuating Intestinal Oxidative Stress and NLRP3 Inflammasome via Activating Aryl Hydrocarbon Receptor Through Remodeling the Gut Microbiome and Metabolites.” Redox Biology 85: 103746. 10.1016/j.redox.2025.103746 PMC12271804 40602277

[22] Wang, Kai , Zhiwei Zhang , Jing Hang , Jia Liu , Fusheng Guo , Yong Ding , Meng Li , et al. 2023. “Microbial‐Host‐Isozyme Analyses Reveal Microbial DPP4 as a Potential Antidiabetic Target.” Science 381: eadd5787. 10.1126/science.add5787 37535747

[23] Wang, Juanjuan , Ningning Zhu , Xiaomin Su , Yunhuan Gao , and Rongcun Yang . 2023. “Gut‐Microbiota‐Derived Metabolites Maintain Gut and Systemic Immune Homeostasis.” Cells 12: 793. 10.3390/cells12050793 PMC10000530 36899929

[24] Xu, Tingting , Dan Fang , Tianqi Xu , Xiuying Tao , Zhiqiang Wang , and Yuan Liu . 2025. “Exercise‐Driven Gut Microbiota Alterations Enhance Colonization Resistance Against Methicillin‐Resistant *Staphylococcus aureus* .” Cell Reports 44: 115424. 10.1016/j.celrep.2025.115424 40080501

[25] Liu, Yuan , Kangni Yang , Yuqian Jia , Jingru Shi , Ziwen Tong , Dan Fang , Bingqing Yang , et al. 2021. “Gut Microbiome Alterations in High‐Fat‐Diet‐Fed Mice Are Associated With Antibiotic Tolerance.” Nature Microbiology 6: 874–884. 10.1038/s41564-021-00912-0 34017107

[26] Wypych, Tomasz P , Lakshanie C Wickramasinghe , and Benjamin J Marsland . 2019. “The Influence of the Microbiome on Respiratory Health.” Nature Immunology 20: 1279–1290. 10.1038/s41590-019-0451-9 31501577

[27] Dang, Anh Thu , and Benjamin J. Marsland . 2019. “Microbes, Metabolites, and the Gut‐Lung Axis.” Mucosal Immunology 12: 843–850. 10.1038/s41385-019-0160-6 30976087

[28] Wang, Tao , Yanling Wang , and Yushi Yao . 2024. “Gut Microbe Guides Alveolar Macrophages to Fight Flu.” Cell Host & Microbe 32: 296–298. 10.1016/j.chom.2024.02.007 38484707

[29] Song, Qian , Xinpei Li , Qing Li , Shijie Shang , Shuling Ma , Zijun Zhai , Fengxin Sun , et al. 2025. “ *Bifidobacterium Animalis* Suppresses Non‐Small Cell Lung Cancer Progression and Modulates Tumor Immunity Through Indole‐3‐Acetic Acid.” Cell Reports 44: 116132. 10.1016/j.celrep.2025.116132 40802513

[30] Shen, Xian , Zhaocong Yang , Qiang Wang , Xu Chen , Qihui Zhu , Zhi Liu , Nishant Patel , Xingyin Liu , and Xuming Mo . 2024. “ *Lactobacillus plantarum* L168 Improves Hyperoxia‐Induced Pulmonary Inflammation and Hypoalveolarization in a Rat Model of Bronchopulmonary Dysplasia.” npj Biofilms and Microbiomes 10: 32. 10.1038/s41522-024-00504-w PMC10980738 38553470

[31] Fan, Lijuan , Bingnan Liu , Youxia Wang , Bin Tang , Tianqi Xu , Jian Fu , Chuanlong Wang , et al. 2024. “Intestinal *Lactobacillus murinus*‐Derived Small RNAs Target Porcine Polyamine Metabolism.” Proceedings of the National Academy of Sciences of the United States of America 121: e2413241121. 10.1073/pnas.2413241121 PMC11474053 39361652

[32] Ye, Xianwen , Zixuan Huang , Yi Li , Mengjiao Wang , Wanyu Meng , Maojian Miao , and Jingdong Cheng . 2024. “Human Tumor Suppressor PDCD4 Directly Interacts With Ribosomes to Repress Translation.” Cell Research 34: 522–525. 10.1038/s41422-024-00962-z PMC11217289 38641729

[33] Jiang, Xiao‐You , Qi‐Qiang Guo , Shan‐Shan Wang , Ran Guo , Yu Zou , Jing‐Wei Liu , Yan‐Ling Feng , et al. 2025. “DNA Damage Response Pathway Regulates Nrf2 in Response to Oxidative Stress.” Science Advances 11: eadu9555. 10.1126/sciadv.adu9555 PMC12293114 40712037

[34] Zhong, Xin , Shiqiang Gong , Linghui Meng , Weifan Yao , Ke Du , Linchi Jiao , Guowei Ma , et al. 2024. “Cordycepin Modulates Microglial M2 Polarization Coupled With Mitochondrial Metabolic Reprogramming by Targeting HKII and PDK2.” Advanced Science 11: e2304687. 10.1002/advs.202304687 PMC11336950 38889331

[35] Zeng, Su‐Ling , Shang‐Zhen Li , Ping‐Ting Xiao , Yuan‐Yuan Cai , Chu Chu , Bai‐Zhong Chen , Ping Li , Jing Li , and E‐Hu Liu . 2020. “Citrus Polymethoxyflavones Attenuate Metabolic Syndrome by Regulating Gut Microbiome and Amino Acid Metabolism.” Science Advances 6: eaax6208. 10.1126/sciadv.aax6208 PMC6941918 31922003

[36] Yao, Huixiang , Yan Shi , Junqing Yuan , Ri Sa , Wei Chen , and Xinjian Wan . 2021. “Matrine Protects Against DSS‐induced Murine Colitis by Improving Gut Barrier Integrity, Inhibiting the PPAR‐α Signaling Pathway, and Modulating Gut Microbiota.” International Immunopharmacology 100: 108091. 10.1016/j.intimp.2021.108091 34474274

[37] Li, Bo , Mingyan Liu , Yu Wang , Shiqiang Gong , Weifan Yao , Wenshuai Li , Hua Gao , and Minjie Wei . 2020. “Puerarin Improves the Bone Micro‐Environment to Inhibit OVX‐Induced Osteoporosis via Modulating SCFAs Released By the Gut Microbiota and Repairing Intestinal Mucosal Integrity.” Biomedicine & Pharmacotherapy 132: 110923. 10.1016/j.biopha.2020.110923 33125971

[38] Huang, Mengnan , Yuanyuan He , Lin Tian , Lu Yu , Qi Cheng , Zhu Li , Lina Gao , Shan Gao , and Chunquan Yu . 2023. “Gut Microbiota‐SCFAs‐Brain Axis Associated With the Antidepressant Activity of Berberine in CUMS Rats.” Journal of Affective Disorders 325: 141–150. 10.1016/j.jad.2022.12.166 36610597

[39] Han, Kai , Fang Xie , Olamide Animasahun , Minal Nenwani , Sho Kitamoto , Yeji Kim , May Thazin Phoo , et al. 2024. “Inulin‐Gel‐Based Oral Immunotherapy Remodels the Small Intestinal Microbiome and Suppresses Food Allergy.” Nature Materials 23: 1444–1455. 10.1038/s41563-024-01909-w PMC11442122 38977883

[40] Mukherjee, Arghya , Cathy Lordan , R Paul Ross , and Paul D Cotter . 2020. “Gut Microbes From the Phylogenetically Diverse Genus Eubacterium and Their Various Contributions to Gut Health.” Gut Microbes 12: 1802866. 10.1080/19490976.2020.1802866 PMC7524325 32835590

[41] Zhang, Ying , Han Jiang , Xiao‐Han Peng , Yan‐Lin Zhao , Xian‐Jie Huang , Kang Yuan , Yi‐Fan Yang , et al. 2025. “Mulberry Leaf Improves Type 2 Diabetes in Mice via Gut Microbiota‐SCFAs‐GPRs Axis and AMPK Signaling Pathway.” Phytomedicine 145: 156970. 10.1016/j.phymed.2025.156970 40527063

[42] Zhang, Kaikai , Jianzheng Yang , Lijian Chen , Jietao He , Dong Qu , Zheng Zhang , Yi Liu , et al. 2023. “Gut Microbiota Participates in Polystyrene Microplastics‐Induced Hepatic Injuries by Modulating the Gut‐Liver Axis.” ACS Nano 17: 15125–15145. 10.1021/acsnano.3c04449 37486121

[43] Davies, Jade , Melinda J Mayer , Nathalie Juge , Arjan Narbad , and Lizbeth Sayavedra . 2024. “ *Bacteroides thetaiotaomicron* Enhances H(2)S Production in *Bilophila wadsworthia* .” Gut Microbes 16: 2431644. 10.1080/19490976.2024.2431644 PMC11610557 39609271

[44] Natividad, Jane M , Bruno Lamas , Hang Phuong Pham , Marie‐Laure Michel , Dominique Rainteau , Chantal Bridonneau , Gregory da Costa , et al. 2018. “ *Bilophila wadsworthia* Aggravates High Fat Diet Induced Metabolic Dysfunctions in Mice.” Nature Communications 9: 2802. 10.1038/s41467-018-05249-7 PMC6052103 30022049

[45] Devkota, Suzanne , Yunwei Wang , Mark W. Musch , Vanessa Leone , Hannah Fehlner‐Peach , Anuradha Nadimpalli , Dionysios A. Antonopoulos , Bana Jabri , and Eugene B. Chang . 2012. “Dietary‐Fat‐Induced Taurocholic Acid Promotes Pathobiont Expansion and Colitis in *Il10*‐/‐ Mice.” Nature 487: 104–108. 10.1038/nature11225 PMC3393783 22722865

[46] Liu, Jianjun , Qiulong Yan , Shenghui Li , Juying Jiao , Yiming Hao , Guixin Zhang , Qingkai Zhang , et al. 2024. “Integrative Metagenomic and Metabolomic Analyses Reveal the Potential of Gut Microbiota to Exacerbate Acute Pancreatitis.” npj Biofilms and Microbiomes 10: 29. 10.1038/s41522-024-00499-4 PMC10957925 38514648

[47] Anhê, Fernando F , Jonathan D Schertzer , and André Marette . 2019. “Bacteria to Alleviate Metabolic Syndrome.” Nature Medicine 25: 1031–1033. 10.1038/s41591-019-0516-1 31263287

[48] Huang, Yuyin , Qingya Wang , Xiling Han , Kun Meng , Guohua Liu , Haiou Zhang , Rui Zhang , Hongying Cai , and Peilong Yang . 2025. “Heat‐Inactivated *Lactiplantibacillus plantarum* FRT4 Alleviates Diet‐Induced Obesity via Gut‐Liver Axis Reprogramming.” Foods 14: 2799. 10.3390/foods14162799 PMC12385762 40870712

[49] Qi, Yadong , Jiamin He , Yawen Zhang , Qiwei Ge , Qiwen Wang , Luyi Chen , Jilei Xu , et al. 2023. “Heat‐Inactivated *Bifidobacterium adolescentis* Ameliorates Colon Senescence through Paneth‐Like‐Cell‐Mediated Stem Cell Activation.” Nature Communications 14: 6121. 10.1038/s41467-023-41827-0 PMC10542354 37777508

[50] Jia, Junpeng , Wenxin Ji , Ningna Xiong , Jian Lin , and Qian Yang . 2025. “Trained Immunity Using Probiotics and Inactivated Pathogens Enhances Resistance to *Salmonella enterica* Serovar Typhimurium Infection by Activating the cGAS‐STING Signal Pathway in Mice and Chickens.” Journal of Advanced Research 79:491–504. 10.1016/j.jare.2025.03.011 PMC12766225 40086629

[51] Zhang, Xiaoming , Ye Wang , Qiyu E , Muhammad Naveed , Xiuli Wang , Yinhui Liu , and Ming Li . 2025. “The Biological Activity and Potential of Probiotics‐Derived Extracellular Vesicles as Postbiotics in Modulating Microbiota‐Host Communication.” Journal of Nanobiotechnology 23: 349. 10.1186/s12951-025-03435-6 PMC12082936 40380331

[52] Hu, Bin , Tian Tian , Xin‐Tao Li , Pei‐Pei Hao , Wei‐Chao Liu , Ying‐Gui Chen , Tian‐Yu Jiang , et al. 2023. “Dexmedetomidine Postconditioning Attenuates Myocardial Ischemia/Reperfusion Injury by Activating the Nrf2/Sirt3/SOD2 Signaling Pathway in the Rats.” Redox Report 28: 2158526. 10.1080/13510002.2022.2158526 PMC9904316 36738240

[53] Wang, Boyang , Defei Kong , Zhiru Yang , Jun Kang , Deyang Sun , Xiumei Duan , Jing Jin , et al. 2025. “Revealing the Mechanisms of Compound Kushen Injection on Oxidative Stress Regulation in the Treatment of Radiation‐Induced Lung Injury.” Engineering 55: 254–268. 10.1016/j.eng.2025.09.018

[54] Xiao, Jun , Shuang Wang , Longlong Chen , Xinyu Ding , Yuanhao Dang , Mingshun Han , Yuxiao Zheng , et al. 2024. “25‐Hydroxycholesterol Regulates Lysosome AMP Kinase Activation and Metabolic Reprogramming to Educate Immunosuppressive Macrophages.” Immunity 57: 1087–1104.e7. 10.1016/j.immuni.2024.03.021 38640930

[55] Shen, Lin , Junchao Yu , Weiqian Chen , Yanran Bi , Zhangyu Yang , Chenying Lu , Chengli Jiang , et al. 2025. “PPP1R3B Suppresses Atherosclerosis by Promoting the M2 Polarization of Macrophages Through Glycogen Metabolic Reprogramming.” Advanced Science 12: e06345. 10.1002/advs.202506345 PMC12591178 40984828

[56] Zhao, Rongchuan , Jiao Yang , Yunjiao Zhai , Hong Zhang , Yuanshuai Zhou , Lei Hong , Detian Yuan , et al. 2024. “Nucleophosmin 1 Promotes Mucosal Immunity by Supporting Mitochondrial Oxidative Phosphorylation and ILC3 Activity.” Nature Immunology 25: 1565–1579. 10.1038/s41590-024-01921-x PMC11362010 39103576

[57] Chen, Shiping , Peiling Zhang , Guiqi Zhu , Biao Wang , Jialiang Cai , Lina Song , Jinglei Wan , et al. 2024. “Targeting GSDME‐mediated Macrophage Polarization for Enhanced Antitumor Immunity in Hepatocellular Carcinoma.” Cellular & Molecular Immunology 21: 1505–1521. 10.1038/s41423-024-01231-0 PMC11607431 39496854

[58] Li, Yangguang , Xuanheng Li , Wenqi Wu , Peizhao Liu , Juanhan Liu , Haiyang Jiang , Liting Deng , et al. 2025. “Insights into the Roles of Macrophages in *Klebsiella pneumoniae* Infections: A Comprehensive Review.” Cellular & Molecular Biology Letters 30: 34. 10.1186/s11658-025-00717-7 PMC11948646 40140770

[59] Chen, Ruifei , Ziyi Liu , Poshi Xu , Xinkun Qi , Shangshang Qin , Zhiqiang Wang , and Ruichao Li . 2021. “Deciphering the Epidemiological Characteristics and Molecular Features of *bla* _KPC‐2_‐ or *bla* _NDM‐1_‐Positive *Klebsiella pneumoniae* Isolates in a Newly Established Hospital.” Frontiers in Microbiology 12: 741093. 10.3389/fmicb.2021.741093 PMC8631570 34858362

[60] Wu, Yingcheng , Shuaixi Yang , Jiaqiang Ma , Zechuan Chen , Guohe Song , Dongning Rao , Yifei Cheng , et al. 2022. “Spatiotemporal Immune Landscape of Colorectal Cancer Liver Metastasis at Single‐Cell Level.” Cancer Discovery 12: 134–153. 10.1158/2159-8290.Cd-21-0316 34417225

