# Supplementary material for: Lactobacillus derived small RNAs protect against Klebsiella pneumoniae infection
Source: Imeta. 2026 Oct 16:e70184. Online ahead of print. doi: 10.1002/imt2.70184 (PMC13673929; doi:10.1002/imt2.70184)
Supplement: Supplementary file 1 [file IMT2-9999-e70184-s001.docx]

**Supporting information to**

***Lactobacillus* derived small RNAs protect against *Klebsiella pneumoniae* infection**

**Running title：***Lactobacillus* small RNAs against *Klebsiella pneumoniae* infection

Tingting Xu^1^, Xiuying Tao^1^, Dan Fang^1^, Haijie Zhang^1,2^, Zhiqiang Wang^1,2,3^, Yuan Liu^1,2,3^*

^1^Jiangsu Co-innovation Center for Prevention and Control of Important Animal Infectious Diseases and Zoonoses, College of Veterinary Medicine, Yangzhou University, Yangzhou 225009, China

^2^Jiangsu Key Laboratory of Zoonosis, Yangzhou University, Yangzhou 225009, China

^3^Jiangsu Interdisciplinary Center for Zoonoses and Biosafety, Yangzhou University, Yangzhou 225009, China

*Correspondence: liuyuan2018@yzu.edu.cn (Yuan Liu)

**Supplementary figures**

**Figure S1** **Gut microbiota driven by natural products enhances lung resistance against CRKP infection, related to Figure 1.** (A) Representative images of HE-stained lung sections from Group C in the natural product supplementation model (scale bars, 100 μm). (B-D) Statistical analyses of body weight (B), lung weight (C), and lung weight index (D) in the FMT model (*n* = 6). Lung weight index, the ratio of lung weight to body weight. (E) Concentrations of the pro-inflammatory factor (IL-6) in the FMT model (*n* = 6). (F) Representative images of clinical morphological observations of the lungs in the FMT model. (G-I) Statistical analyses of mucus thickness (G), crypt depth (H), and the V/C ratio (I) in the duodenum in the FMT model (*n* = 3). V/C, the ratio of villus height to crypt depth. (J) Representative images of HE-stained colon sections in the FMT model (scale bars, 200 μm). (K) Establishment of a gut microbiota clearance model in mice. Natural products were administered for five consecutive days, followed by intragastric administration of antibiotics-containing drinking water to eliminate gut microbiota, and then infection with CRKP was performed (*n* = 4). (L) Bacterial loads of CRKP in the lungs of the (K) model (*n* = 4). (M-O) Concentrations of inflammatory factors in the (K) model (*n* = 4 to 12). (P) Representative images of HE-stained lung sections in the (K) model (scale bars, 200 μm). Data are presented as the mean ± SEM. *n* values represent the number of biological replicates. One-way ANOVA with Tukey’s post hoc test was used to calculate *p* values in (B-E), (G-I), and (L-O). **p* < 0.05, ***p* < 0.01, ****p* < 0.001, and *****p* < 0.0001. ns, not significant.

**Figure S2 Changes in the structure of the gut microbiota in the natural product supplementation model, related to Figure 2.** (A-C) Alpha diversity analysis, determined by chao1 (A), coverage (B), and simpson (C) in mice. (D and E) Unweighted UniFrac distance-based PCoA (D) and NMDS (E) analysis of gut microbiota in C group (red), E group (blue), and N group (yellow) in mice. (F) Top 5 genera in abundance with significant differences between E (E1-E6) groups and N1 group in mice. (G) Top 5 genera in abundance with significant differences between E (E1-E6) groups and the N2 group in mice. (H) Top 5 genera in abundance with significant differences between E (E1-E6) groups and the N3 group in mice. Data in (F), (G), and (H) are presented as the mean ± SEM. Kruskal-Wallis test with Dunn’s post hoc test was used to calculate *p* values in (A), (B), and (C). Mann-Whitney U test was used to calculate *p* values in (F), (G), and (H). Biological repetitions were performed. **p* < 0.05, ***p* < 0.01, ****p* < 0.001, and *****p* < 0.0001. ns, not significant.

**Figure S3** **Analysis of gut microbiota in the natural product supplementation model, related to Figure 2J.** (A) Top 5 genera in abundance with significant differences between E (E1-E6) groups and N4 group in mice. (B) Top 5 genera in abundance with significant differences between E (E1-E6) groups and the N5 group in mice. (C) Top 5 genera in abundance with significant differences between E (E1-E6) groups and the N6 group in mice. (D) Top 5 genera in abundance with significant differences between E (E1-E6) groups and the Con group in mice. Data are presented as the mean ± SEM. Mann-Whitney U test was used to calculate *p* values. Biological repetitions were performed. **p* < 0.05, ***p* < 0.01, ****p* < 0.001, and *****p* < 0.0001. ns, not significant.

**Figure S4 Stable colonization of *Lactobacillus murinus* enhances intestinal barrier function, related to Figure 3.** (A) Number of copies of *L. murinus* in feces in the FMT model (*n* = 3). (B) Statistical analyses of duodenal villus height and crypt depth in the *L. murinus* supplementation model (*n* = 3). (C) Representative images of clinical morphological observations of the lungs in mice. (D) Statistical analyses of lung weight and lung weight index (*n* = 6). Lung weight index, the ratio of lung weight to body weight. (E) Representative images of TUNEL staining in the lungs in the *L. murinus* supplementation model (scale bars, 50 μm). (F) Concentrations of the pro-inflammatory (IL-6) factor in mice (*n* = 6). Data are presented as the mean ± SEM. *n* values represent the number of biological replicates. One-way ANOVA with Tukey’s post hoc test was used to calculate *p* values in (A). Unpaired two-tailed Student's *t* test was used to calculate *p* values in (B), (D), and (F). **p* < 0.05, ***p* < 0.01, ****p* < 0.001, and *****p* < 0.0001. ns, not significant.

**Figure S5 *Lactobacillus* protects against CRKP infection, related to Figure 4.** (A) Bacterial load of CRKP in mouse lungs after supplementation with HK-*L. m*, HK-*L. r*, HK-*L. j*, and HK-*L. t* (*n* = 6 to 12). (B-D) Concentrations of pro-inflammatory (IL-22, IL-1β, and IL-6) and anti-inflammatory (IL-4, IL-10, and TGF-β) factors in mice lungs (*n* = 3). (E and F) Bacterial load of CRKP in mouse lungs after supplementation with UL-*L. m*^DNA^, UL-*L. m*^RNA^, and UL-*L. m*^Protein^ (*n* = 6). (G) Polyacrylamide gel electrophoresis of protein from HK-*L. m*^Cells^, HK-*L. m*^SNF^, and UL-*L. m*. (H and I) Absorbance values of DNA (H) and RNA (I) for HK-*L. m*^Cells^, HK-*L. m*^SNF^, and UL-*L. m*. (J) Quantitative analysis of MVs internalization by macrophages in Figure 4K. (K) Representative images of lung HE-stained sections in mice (scale bars, 200 μm). (L) Bacterial load of CRKP in mouse lungs (*n* = 16). Related to figure 4L. Data are presented as the mean ± SEM. *n* values represent the number of biological replicates. One-way ANOVA with Tukey’s post hoc test was used to calculate *p* values for three or more groups. Mann-Whitney U test was used to calculate *p* values in (L). **p* < 0.05, ***p* < 0.01, ****p* < 0.001, and *****p* < 0.0001. ns, not significant.

**Figure S6** **sRNA analysis of bacteria and lungs, related to Figure 5.** (A and B) Relative expression ratio of sRNAs in bacterial samples (MVs and HK-*L. m*) from sRNA sequencing results. TPM, transcripts per million. (C and D) Relative expression ratio of sRNAs in lung samples (Con and HK-*L. m* groups) from sRNA sequencing results. TPM, transcripts per million. (E) Schematic diagram of intragastric administration of *L. murinus* in mice (*n* = 3). (F) Copy number and particle count of MVs in lungs. (G and H) Digital PCR analysis of mim-miR-21a-5p in MVs. (I) Copy number of mim-miR-21a-5p in lungs. (J) Representative images of immunofluorescence of MVs loaded with mim-miR-21a-5p (scale bars, 10 μm). (K) Quantitative analysis of MVs and mim-miR-21a-5p internalization by macrophages in Figure 5H. (L) Relative expression of mim-miR-21a-5p in macrophages transfected with mim-miR-21a-5p mimic (*n* = 3). (M) Relative expression of mim-miR-21a-5p in macrophages after co-incubation with MVs loaded with a mim-miR-21a-5p inhibitor (*n* = 3). (N) Relative expression of mim-miR-21a-5p in MVs from *L. reuteri, L. taiwanensis, L. johnsonii,* and *L. murinus* (*n* = 3). (O) Gel electrophoresis of PCR amplification products of mim-miR-21a-5p from the MVs of *L. m*, *L. r*, *L. t,* and *L. j*. Data are presented as the mean ± SEM. *n* values represent the number of biological replicates. Unpaired two-tailed Student's *t* test was used to calculate *p* values between two groups. One-way ANOVA with Tukey’s post hoc test was used to calculate *p* values for three or more groups. Biological repetitions were performed. **p* < 0.05, ***p* < 0.01, ****p* < 0.001, and *****p* < 0.0001. ns, not significant.

 **Figure S7 *L. m*, HK-*L. m*, and** ***L. m*-MVs exhibit no direct inhibitory effect on CRKP *in vitro*, related to Figure 5.** (A) Establishment of a chronic CRKP infection model (*n* = 6). (B and C) Bacterial loads of CRKP in the lung, liver, and kidney of (A). (D and E) Bacterial loads of CRKP at different time points after co-incubation with L. m at varying ratios in LB (D) or MRS (E) medium (*n* = 3). (F) MIC values of HK-*L. m* and *L. m*-MVs against CRKP. (G) Growth curves of MVs co-cultured with CRKP at low, medium, and high doses (*n* = 3). (H) Volcano plot of significantly differentially expressed genes (DEGs) in macrophages between the Con and MVs groups. Purple dots, upregulated; Gray dots, not significant; Pink dots, down-regulated. *p* value ≤ 0.05. (I) GO analysis of significantly DEGs in macrophages between the Con and MVs groups. (J) GO enrichment analysis of sRNAs in lung samples (Con and HK-*L. m* groups). Data are presented as the mean ± SEM. *n* values represent the number of biological replicates. One-way ANOVA with Tukey’s post hoc test was used to calculate *p* values in (C). Unpaired two-tailed Student's *t* test was used to calculate *p* values in (B). **p* < 0.05, ***p* < 0.01, ****p* < 0.001, and *****p* < 0.0001. ns, not significant.

**Figure S8 *PDCD4* suppression sustains OXPHOS via activating the *Nrf2*/*SOD2* pathway, related to Figure 6.** (A) Schematic diagram of mim-miR-21a-5p target genes predicted using miRWalk and TargetScan software. (B) mRNA relative expression of *PTEN* and *PDCD4* in macrophages treated with MVs (*n* = 3). (C and D) mRNA and protein expression levels of PDCD4 in macrophages after co-incubation with MVs loaded with mim-miR-21a-5p inhibitor (*n* = 3). (E) mRNA expression levels of *SOD2* and *Nrf2* in macrophages treated with MVs (*n* = 3). (F and G) mRNA expression levels of *PDCD4*, *SOD2,* and *Nrf2* after *Pdcd4* knockout in macrophages (*n* = 3). (H) Bacterial load of CRKP in macrophages (*n* = 6). (I and J) Absolute abundance and relative abundance of PDCD4 (I) and SOD2 (J) in 20 clusters from single-cell transcriptomic sequencing. Cluster17, macrophages. (K and L) Concentrations of Complex III (K), Complex IV (K), and SDH (L) in macrophages treated with MVs (*n* = 3 to 4). SDH, succinate dehydrogenase. (M) The luminescence value of JC-1 aggregates and JC-1 monomers in macrophages treated with MVs (*n* = 10). CCCP, Positive. (N and O) The luminescence value of ATP, ADP, and ROS in macrophages treated with MVs (*n* = 3 to 6). Positive Rosup, positive control. Data are presented as the mean ± SEM. *n* values represent the number of biological replicates. One-way ANOVA with Tukey’s post hoc test was used to calculate *p* values in (D), (F, G, H), (M), and (O). Unpaired two-tailed Student's *t* test was used to calculate *p* values in (B), (E), (K, L), and (N). **p* < 0.05, ***p* < 0.01, ****p* < 0.001, and *****p* < 0.0001. ns, not significant.

**Figure S9 OXPHOS-sustained M2 macrophage polarization enhances host resistance to CRKP infection, related to Figure 6.** (A) mRNA expression levels of *Ym1*, *CD206*, *Arg-1,* and *IL-10* in macrophages treated with MVs (*n* = 3). (B) *TNF-α* mRNA expression levels in macrophages treated with MVs (*n* = 3). (C) The level of CD86 in macrophages treated with MVs was measured by flow cytometry. MFI, mean fluorescence intensity. LPS, Positive. (D) The luminescence value of ROS in macrophages treated with MVs and rotenone (*n* = 6). Positive Rosup, positive control. (E) The ΔΨm changes in macrophages treated with MVs and rotenone (*n* = 6). ΔΨm, JC-1 aggregates/monomer. CCCP, Positive. (F) The luminescence value of JC-1 aggregates and JC-1 monomer in macrophages treated with MVs and rotenone (*n* = 6). CCCP, Positive. (G and H) Western blots showing Arg-1 and iNOS expression in macrophages treated with MVs and rotenone (*n* = 3). (I) mRNA expression levels of *IL-10*, *Arg-1, Ym1,* and *TNF-α* in macrophages treated with MVs and rotenone (*n* = 3). (J and K) t-SNE distribution of M2 macrophages (cluster 17) in the Con (J) and MVs (K) groups. Green, M2 macrophages. (L) Proportion of M2 macrophage marker genes in the clusters. (M-O) Bacterial load of C10 (M), C14 (N), and C18 (O) in mouse lungs (*n* = 6). Mice received intragastric supplementation of HK-*L. m*, HK-*L. r*, HK-*L. t*, HK-*L. j,* and *L. m*-MVs for 7 days, followed by intranasal challenge with different CRKP strains. Lungs were collected at 6 h post‑infection for plate counting of bacterial burden. (P) The radar chart illustrates the enrichment profiles of mim-miR-21a-5p downstream pathways, oxidative phosphorylation-related indicators, and macrophage M2 polarization-related indicators in the CON (blue) and MVs (orange) groups. Each axis represents an individual indicator, with its value presented on a log10 scale. The distance extending from the center to the periphery on each axis corresponds to the magnitude of the indicator's value. Data are presented as the mean ± SEM. *n* values represent the number of biological replicates. One-way ANOVA with Tukey’s post hoc test was used to calculate *p* values in (D, E, F), (H, I), and (M, N, O). Unpaired two-tailed Student's *t* test was used to calculate *p* values in (A) and (B). **p* < 0.05, ***p* < 0.01, ****p* < 0.001, and *****p* < 0.0001. ns, not significant.
